# Hypoxia‐related THBD ^+^ macrophages as a prognostic factor in glioma: Construction of a powerful risk model

**DOI:** 10.1111/jcmm.18393

**Published:** 2024-05-29

**Authors:** Weichun Tang, Juntao Du, Lin Li, Shangshang Hu, Shuo Ma, Mengtong Xue, Linlin Zhu

**Affiliations:** ^1^ Blood Transfusion Department The Third People's Hospital of Bengbu Bengbu China; ^2^ Department of Rehabilitation Medicine The First Affiliated Hospital of Bengbu Medical College Bengbu China; ^3^ Anhui Key Laboratory of Tissue Transplantation Bengbu Medical College Bengbu China; ^4^ Medical School of Southeast University Nanjing China; ^5^ School of Medical Technology Xinxiang Medical University Xinxiang China

**Keywords:** gliomas, hypoxia, macrophage, prognostic, THBD

## Abstract

Glioma is a prevalent malignant tumour characterized by hypoxia as a pivotal factor in its progression. This study aims to investigate the impact of the most severely hypoxic cell subpopulation in glioma. Our findings reveal that the THBD^+^ macrophage subpopulation is closely associated with hypoxia in glioma, exhibiting significantly higher infiltration in tumours compared to non‐tumour tissues. Moreover, a high proportion of THBD^+^ cells correlates with poor prognosis in glioblastoma (GBM) patients. Notably, THBD^+^ macrophages exhibit hypoxic characteristics and epithelial‐mesenchymal transition features. Silencing THBD expression leads to a notable reduction in the proliferation and metastasis of glioma cells. Furthermore, we developed a THBD^+^ macrophage‐related risk signature (THBDMRS) through machine learning techniques. THBDMRS emerges as an independent prognostic factor for GBM patients with a substantial prognostic impact. By comparing THBDMRS with 119 established prognostic features, we demonstrate the superior prognostic performance of THBDMRS. Additionally, THBDMRS is associated with glioma metastasis and extracellular matrix remodelling. In conclusion, hypoxia‐related THBD^+^ macrophages play a pivotal role in glioma pathogenesis, and THBDMRS emerges as a potent and promising prognostic tool for GBM, contributing to enhanced patient survival outcomes.

## 1 INTRODUCTION

Gliomas are intracranial tumours that typically originate from glial cells or their precursor cells in the brain.^1^, ^2^ The incidence of adult gliomas is 6.94 per 100,000, varying depending on age, gender, race and geographic location.^3^ The 2016 World Health Organization (WHO) classification of central nervous system tumours divides gliomas into low‐grade tumours (LGG, WHO I–II) and high‐grade tumours (HGG WHO III–IV), with glioblastoma (GBM) being classified as WHO Grade IV.^4^ The median age at diagnosis varies by histological subtype, with pilocytic astrocytoma being more common in children and adolescents, low‐grade oligodendrogliomas peaking in the 30s and 40s, and GBMs predominantly affecting patients over 50 years old.^5^ The overall survival (OS) rate for WHO Grade 4 is the poorest, with a median survival time of approximately 8 months.^6^ Current standard treatments for gliomas encompass surgical resection, radiotherapy and chemotherapy; however, their efficacy is frequently constrained by the aggressive and heterogeneous characteristics of gliomas.^7^, ^8^ Thus, there is a pressing need for the identification of molecular biomarkers that are more precise, with prognostic and therapeutic implications, to enhance the clinical management of gliomas.

Over the recent years, the tumour microenvironment (TME) has garnered significant attention in research aimed at unravelling tumour biology and advancing drug development. The TME is a sophisticated system encompassing various non‐cellular and cellular elements, such as immune cells, endothelial cells, cancer‐associated fibroblasts (CAFs) and cytokines.^9^, ^10^ Accumulating evidence underscores the critical role of interactions among diverse stromal components within the TME and tumour cells, influenced by various factors, in tumour growth and metastasis.^11^ Hypoxia, a hallmark feature of the TME present in nearly all solid tumours, is a key factor that can modulate tumour behaviour. Various tumour characteristics, including uncontrolled proliferation, microvascular abnormalities, altered diffusion geometry and tumour‐associated anaemia, contribute to the development of hypoxia.^12^ Hypoxia exerts a regulatory effect on tumour proliferation, angiogenesis, invasiveness, metastasis and resistance to radiotherapy.^13^ Furthermore, studies have demonstrated that hypoxia can enhances tumour cell resistance to immune‐mediated attacks and evasion of immune surveillance.^14^ Hypoxia‐inducible factors (HIF), pivotal mediators of hypoxia signalling, control a panel of genes that modulate tumour immunity under hypoxic conditions.^15^ Research suggests that hypoxic regions within tumours exhibit heightened infiltration of myeloid‐derived suppressor cells (MDSCs), tumour‐associated macrophages (TAMs) and T regulatory cells (Treg).^16^ Prolonged hypoxia can activate HIF‐1a, which suppresses the activity of cytotoxic lymphocytes, such as NK cells and CD8^+^ T cells, thereby impeding their effectiveness in combating tumours.^17^ Moreover, compelling evidence suggests that hypoxia strongly influences the induction of hif‐1a‐dependent PD‐L1 expression in tumour cells, macrophages and dendritic cells, which serves as a crucial mechanism for obstructing antitumor immunity in cancer cells.^9^, ^18^ Therefore, there is a need to investigate innovative hypoxia‐related biomarkers and understand their roles in gliomas, as this could aid in the diagnosis and prognosis of glioma patients.

Single‐cell RNA sequencing (scRNA‐seq) is a methodology utilized to unveil stochastic heterogeneity within cell populations and to map developmental ‘trajectories’ of immune cells, revealing novel and distinct immune cell subsets.^19^ This approach effectively addresses the limitations of traditional RNA sequencing methods. In contrast, the conventional population‐based RNA sequencing method (bulk RNA‐seq) plays a pivotal role in deciphering genome‐wide transcriptome variation, but may mask transcriptional patterns within specific subpopulations due to the dominance of certain cell types or states.^20^, ^21^ While the combination of scRNA‐seq and bulk RNA‐seq has been widely applied in studies focusing on tumour immunology,^21^ research utilizing these methods in the context of gliomas is currently limited. Additionally, machine learning serves as an indispensable tool, employing sophisticated algorithms to automatically analyse large and heterogeneous datasets, uncover distinct patterns and predict potential issues.^21^ With the advancement of bioinformatics, machine learning has become a standard tool for evaluating the risk and treatment requirements of individual patients. However, relying solely on a single machine learning algorithm may limit its capacity to offer optimal clinical care, as it may not fully leverage the available data. An integrated approach based on multiple algorithms could potentially establish a consensus model for prognostic prediction, thereby allowing for a more personalized method of clinical decision‐making.

In this study, we investigated the relationship between hypoxia, gliomas progression and prognosis by utilizing bulk, single‐cell and spatial transcriptomic sequencing, in conjunction with high‐dimensional weighted gene co‐expression network analysis (hdWGCNA). The aim was to identify the cellular subpopulation most strongly associated with hypoxia. Subsequently, a risk prediction signature was developed and validated through the integration of 101 algorithmic combinations derived from 10 distinct machine learning algorithms. This predictive signature exhibited exceptional prognostic robustness. Functional analyses unveiled that the prognostic model was intimately connected to gliomas metastasis and extracellular matrix remodelling. Our findings show promise for optimizing precision therapy and improving the clinical prognosis of gliomas cancer patients.

## 2 MATERIALS AND METHODS

The detailed content of Materials and methods can be found in Appendix 1 of the supplementary materials.

## 3 RESULTS

### 3.1 The malignancy of gliomas is associated with hypoxia

**FIGURE 1:**
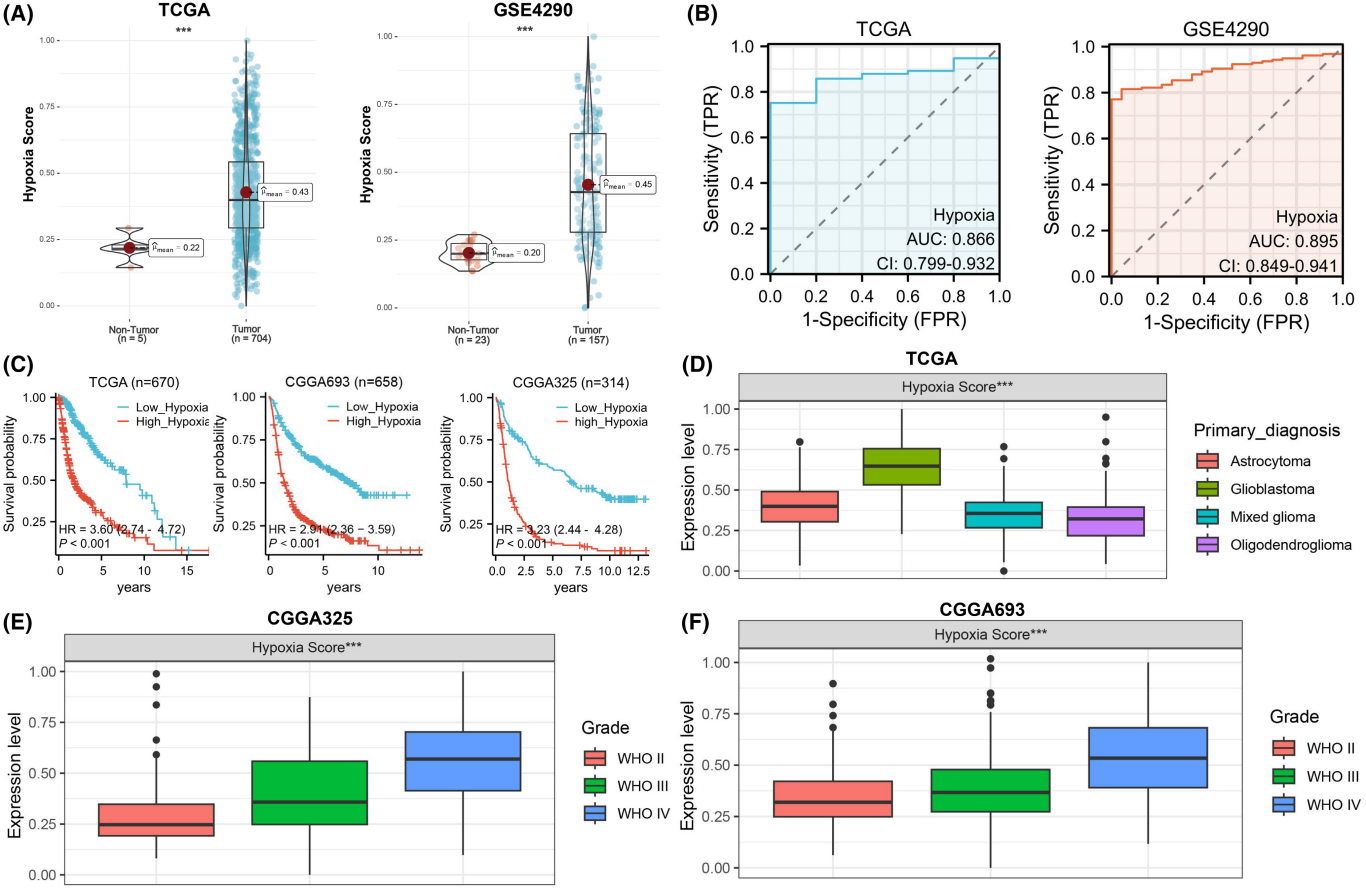
Hypoxia as a characteristic of gliomas. (A) Comparison of hypoxia scores between tumour patients and matched controls in the TCGA and GES4290 datasets. (B) ROC curve analysis determining the AUC values for hypoxia in distinguishing gliomas patients from matched controls. (C) Kaplan–Meier curve analysis of the survival outcomes of tumour patients in the high and low hypoxia score groups. (D) Hypoxia scores for different types of gliomas in the TCGA database. (E, F) Hypoxia scores for gliomas patients at different stages in the CGGA325 and CGGA693 databases.

To investigate the relationship between gliomas progression and hypoxia, we initially evaluated individual hypoxia scores for tumour patients and matched controls in the TCGA and GES4290 datasets. Our findings indicated that gliomas exhibited substantially elevated levels of hypoxia compared to the control group (Figure 1A), with respective AUC values of 0.866 and 0.895 for discriminating between tumour patients and matched controls (Figure 1B). Additionally, in three additional validation datasets, the Kaplan–Meier analysis demonstrated a more favourable prognosis in the hypoxia‐low group relative to the hypoxia‐high group (Figure 1C). Subsequent hypoxia scoring of various types of glioma patients in the TCGA dataset revealed that individuals with the highest malignancy grade glioma, glioblastoma multiforme (GBM), exhibited the highest hypoxia scores, which escalated with disease severity (Figure 1D‐F). These results suggest that hypoxia is a characteristic of gliomas and is a factor associated with disease progression and poor prognosis in gliomas patients.

### 3.2 Assessment of hypoxia‐associated features in gliomas scRNA‐seq data

**FIGURE 2:**
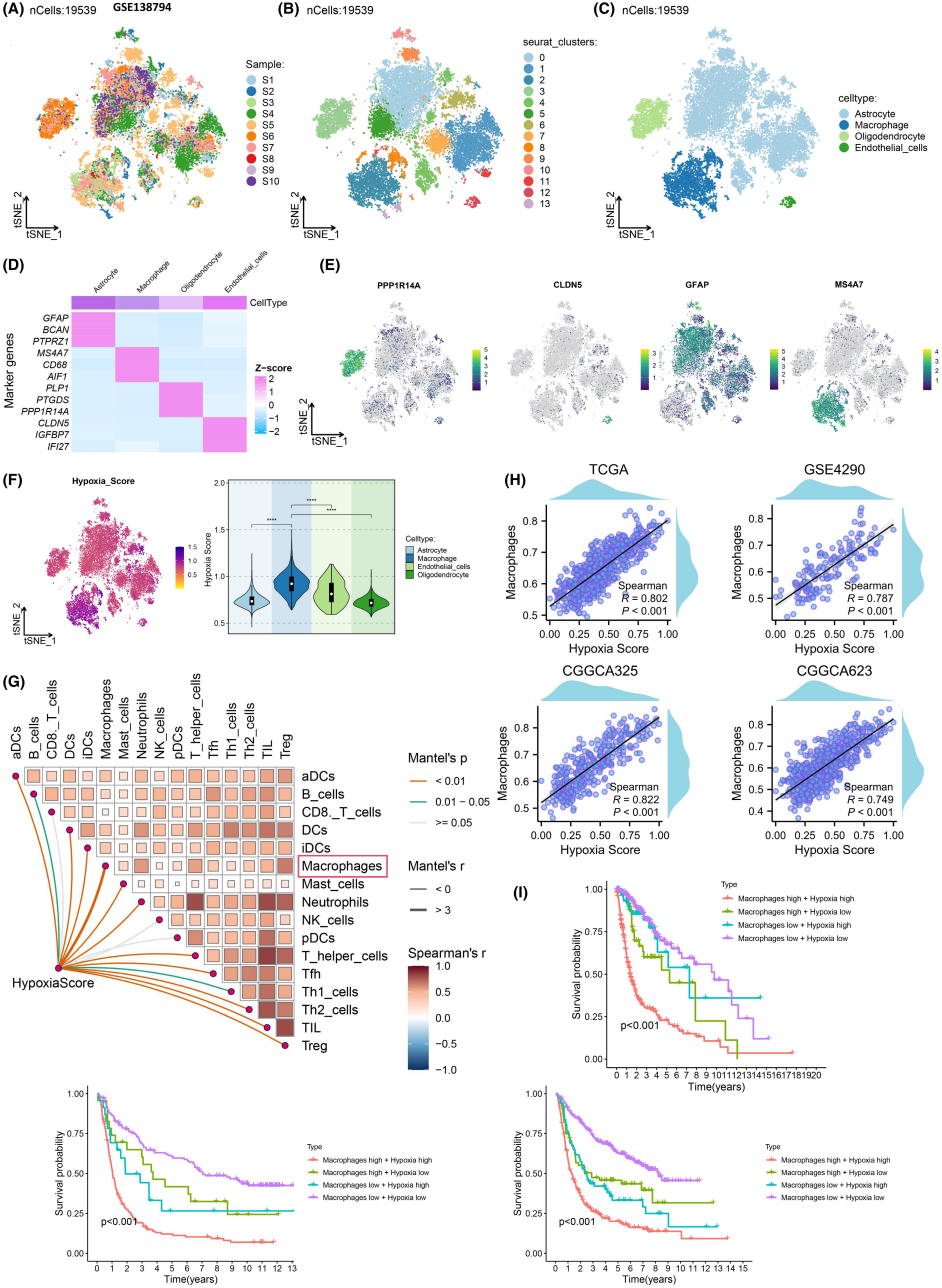
Analysis of hypoxia features in gliomas patients using single‐cell sequencing. (A) Analysis of the GSE138794 dataset using scRNA‐seq identified a total of 19,539 cells. (B) t‐SNE algorithm plotted 14 different cell clusters. (C) Four immune cell populations were identified based on canonical markers. (D, E) Heatmap and t‐SNE visualization of the expression and distribution of cell markers before and after integration. (F) t‐SNE plot showing hypoxia scores for each cell type, with darker purple indicating higher scores. (G) Heatmap demonstrating the correlation between various cell types and hypoxia based on bulk‐RNA sequencing. (H) Correlation between hypoxia scores and macrophage infiltration in TCGA, GSE4290, CGGCA3225 and CGGCA623 databases. (I) Kaplan–Meier curve analysis of survival outcomes in different groups of gliomas patients.

To investigate the heterogeneity of gliomas and assess variations in hypoxia levels between cells within the tumour and adjacent cells, scRNA‐seq data from 10 glioma samples in the GSE138794 dataset were analysed (Figure 2A). Following rigorous quality control measures to exclude low‐quality cells, a total of 19,539 cells were retained. Dimensionality reduction and unsupervised clustering were conducted using t‐SNE, leading to the identification of 14 distinct cell clusters (Figure 2B). By leveraging known markers, four cell types were distinguished within the 14 cell clusters (Figure 2C), and the expression profiles and spatial distribution of cell markers were visualized through heatmaps and t‐SNE plots: Astrocytes (marker: GFAP), macrophages (marker: MS4A7), oligodendrocytes (marker: PPP1R14A) and endothelial cells (marker: CLDN5) (Figure 2D,E). Subsequently, a hypoxia score was computed for gene sets in the single‐cell dataset based on the expression levels of hypoxia‐related genes, as depicted in Figure 2F, highlighting relatively elevated hypoxia scores in macrophages. Moreover, in bulk sequencing data, the association between hypoxia and macrophages remained significant, with macrophage infiltration intensifying with increasing hypoxia scores (Figure 2G,H). Survival analysis integrating macrophage infiltration and hypoxia levels revealed that the macrophages high+ hypoxia high subgroup exhibited the poorest survival outcomes compared to other groups (Figure 2I). These findings suggest that macrophages exhibit heightened hypoxia scores across different glioma subgroups and that their infiltration levels are positively correlated with hypoxia.

### 3.3 Macrophages polarize towards THBD ^+^ macrophages as hypoxia levels deepen

**FIGURE 3:**
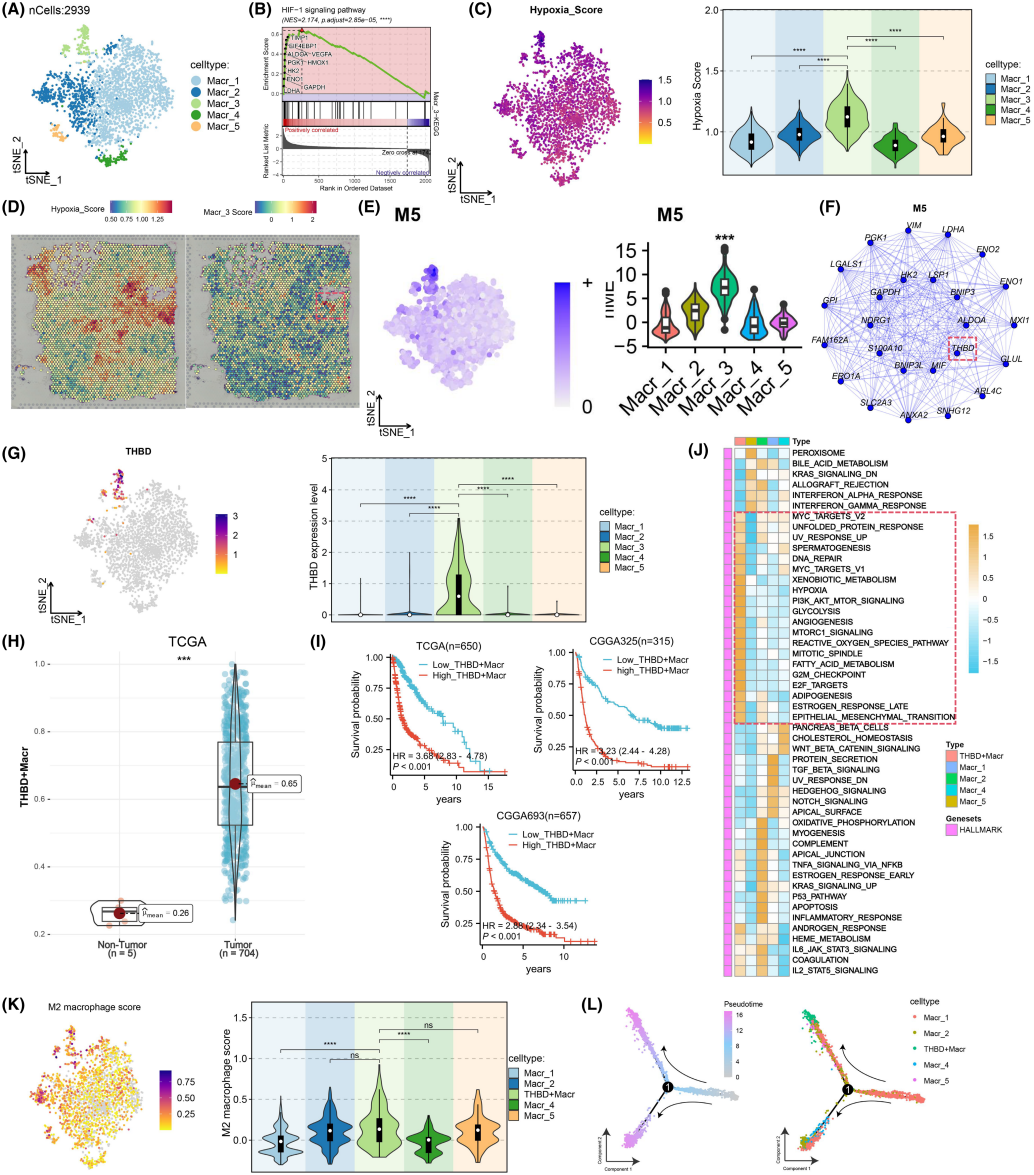
THBD gene as a key gene for macrophage hypoxia. (A) t‐SNE plot displaying five subgroups of macrophages. (B) GSEA pathway analysis of differentially expressed genes in the Macr_3 subgroup. (C) t‐SNE plot showing the hypoxia scores for each macrophage type. (D) Spatial transcriptomic analysis of the major hypoxic regions in gliomas and localization of Macr_3 score. (E) t‐SNE plot demonstrating the correlation between M5 and hypoxia. (F) PPI network analysis revealing THBD as a key gene in the M5 module. (G, H) Upregulation of THBD gene in gliomas and Macr_3 subgroup. (I) Kaplan–Meier curve analysis of survival outcomes in high THBD^+^ macrophage group and low THBD^+^ macrophage group in different databases. (J) KEGG pathway analysis of key genes in THBD^+^ macrophages. (K) t‐SNE plot displaying M2 polarization scores for macrophages. (L) Cell trajectory and pseudo‐time analysis of macrophages.

Given the elevated hypoxia scores of macrophages, we conducted clustering analysis to categorize macrophages based on their characteristic gene features, revealing five distinct macrophage subtypes (Figure 3A; Figure S1A). Subsequent KEGG and GSEA pathway enrichment analyses indicated that the differentially expressed genes in the Macr_3 subtype primarily participated in the hypoxia‐linked HIF‐1 signalling pathway^15^ (Figure 3B; Figure S1B). This led us to investigate the hypoxia levels across these subtypes. Our results confirmed our hypothesis, showing that the Macr_3 macrophage subtype exhibited the highest hypoxia score, with hypoxia levels escalating with the extent of infiltration by this subtype (Figure 3C; Figure S1C). Furthermore, we delved into the spatial transcriptomics sequencing data of glioma patients to pinpoint the principal hypoxic regions, revealing that the Macr_3 macrophage subtype score predominated in the core hypoxic areas of gliomas (Figure 3D). Leveraging hdWGCNA, we discerned the primary molecular characteristics of the Macr_3 macrophage subtype. By setting a soft threshold of 1, an unscaled network was established for the Macr_3 subtype, leading to the identification of six gene modules (Figure S2A,B). Notably, module 5 exhibited the highest gene set score within the Macr_3 subtype (Figure 3E; Figure S2C,D). The top 25 core node network of module 5′s co‐expression network is illustrated in Figure 3F, highlighting THBD as the most central node (Figure 3F). Further investigation revealed that THBD was primarily expressed in macrophages (Figure S2E) and exhibited specific expression in the Macr_3 subtype (Figure 3G,H). Consequently, we designated the Macr_3 subtype as THBD^+^ macrophages. Survival analysis across different datasets also indicated a more favourable prognosis in the THBD^+^ macrophages low group (Figure 3I). Pathway analysis unveiled that the predominant genes in the THBD^+^ macrophage subtype were primarily enriched in critical pathways including PI3K_AKT_MTOR_SIGNALLING, MTORC1_SIGNALLING, REACTIVE_OXYGEN_SPECIES_PATHWAY and EPITHELIAL MESENCHYMAL_TRANSITION (Figure 3J). Given that hypoxia can trigger macrophage polarization,^18^ we further evaluated the M2 polarization scores for distinct cell subtypes. The findings demonstrated that THBD^+^ macrophages exhibited elevated M2 polarization scores compared to other subtypes, showing a positive correlation with their infiltration levels (Figure 3K; Figure S2F). Subsequently, trajectory analysis was conducted on the cellular subpopulations within the spatial domain, revealing that hypoxia facilitated macrophage polarization towards an M2 phenotype. Remarkably, with increasing hypoxia levels, different subtypes tended to polarize towards THBD^+^ macrophages (Figure 3L). In summary, our study unveiled a positive correlation between the extent of macrophage infiltration and hypoxia scores, progressively leading to polarization towards THBD^+^ macrophages as hypoxia levels intensified. This novel finding suggests a unique mechanism in gliomas.

### 3.4 THBD ^+^ macrophages promote the proliferation, migration and invasion of malignant tumour cells

**FIGURE 4:**
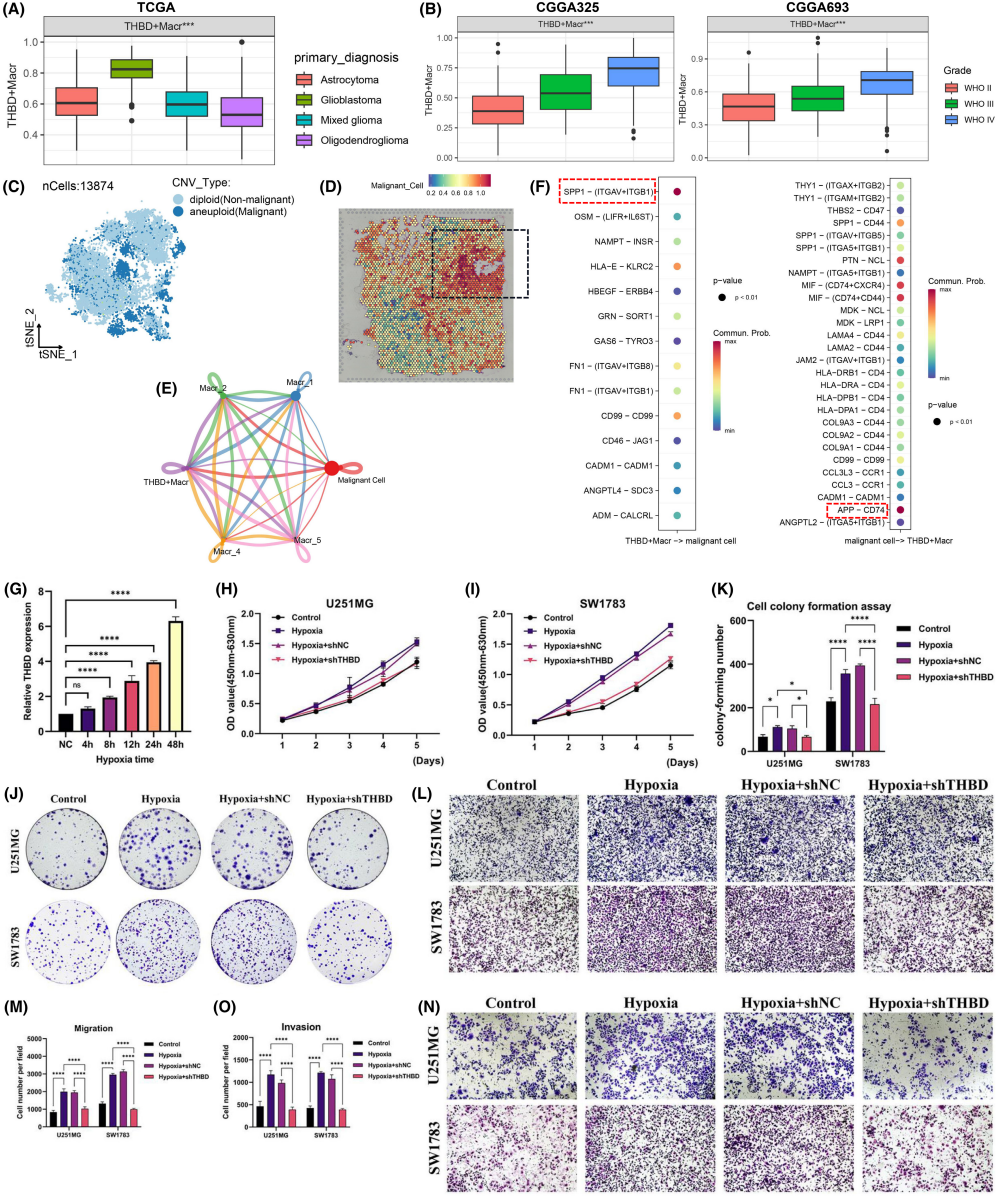
THBD^+^ macrophages promote proliferation, migration and invasion of malignant tumour cells. (A). Expression of THBD molecule in different types of gliomas patients in the TCGA database. (B). Expression of the THBD molecule in gliomas patients at different stages in the CGGA325 and CGGA693 databases. (C, D) t‐SNE plot and spatial transcriptomics display the expression and localization of malignant cells in gliomas. (E) Circular plot illustrating the interaction between different subtypes of macrophages and malignant tumour cells in gliomas. (F) Bubble plot showing ligand‐receptor interactions between THBD^+^ macrophages and malignant tumour cells. (G) Expression of THBD molecule within macrophages in different groups. (H–K) CCK‐8 assay and colony formation experiment validating the proliferation of gliomas cells in different groups. (L–O) Transwell assay validating the migration and invasion of gliomas cells in different groups.

To investigate the impact of THBD^+^ macrophages on gliomas, we initially utilized bulk sequencing data to assess the infiltration level of THBD^+^ macrophages across various glioma types. The findings indicated a notably elevated infiltration level of THBD^+^ macrophages in the most malignant gliomas, with the infiltration score positively correlating with glioma malignancy (Figure 4A,B). Subsequent single‐cell transcriptomic and spatial transcriptomic analyses unveiled the predominant localization of malignant glioma cells within hypoxic core regions (Figure 4C,D). To elucidate the interaction between THBD^+^ macrophages and malignant cells, cell–cell communication analysis was performed, demonstrating the robust communication capacity of THBD^+^ macrophages with malignant tumour cells compared to other subtypes (Figure 4E). Moreover, ligands implicated in the interaction between THBD^+^ macrophages and malignant tumour cells were identified, including SPP1‐(ITGAV+ITGB1) and APP‐CD74 (Figure 4F), suggesting the potential of THBD^+^ macrophages to influence malignant tumour cells through diverse ligands. To validate the functional impact of THBD^+^ macrophages on malignant tumour cells, in vitro experiments were conducted. RT‐qPCR results exhibited a significant upregulation of THBD expression in macrophages following hypoxic treatment, with expression levels escalating with prolonged hypoxic exposure (Figure 4G). Additionally, we treated macrophages differently and cocultured them with two types of GBM malignancy cells (U251MG and SW1783) to assess their effects on tumour cells. The results indicated that the hypoxia group cells exhibited faster proliferation rates, and increased migration and invasion numbers compared to the control group with U251MG and SW1783 cells (control), but these effects could be reversed by knocking down THBD (Figure 4H–O). In summary, the findings suggest a positive correlation between the presence of THBD^+^ macrophages and the aggressiveness of gliomas, with this particular subtype facilitating the proliferation, migration and invasion of malignant cells within gliomas.

### 3.5 Screening for key genes related to hypoxia in THBD ^+^ macrophages

**FIGURE 5:**
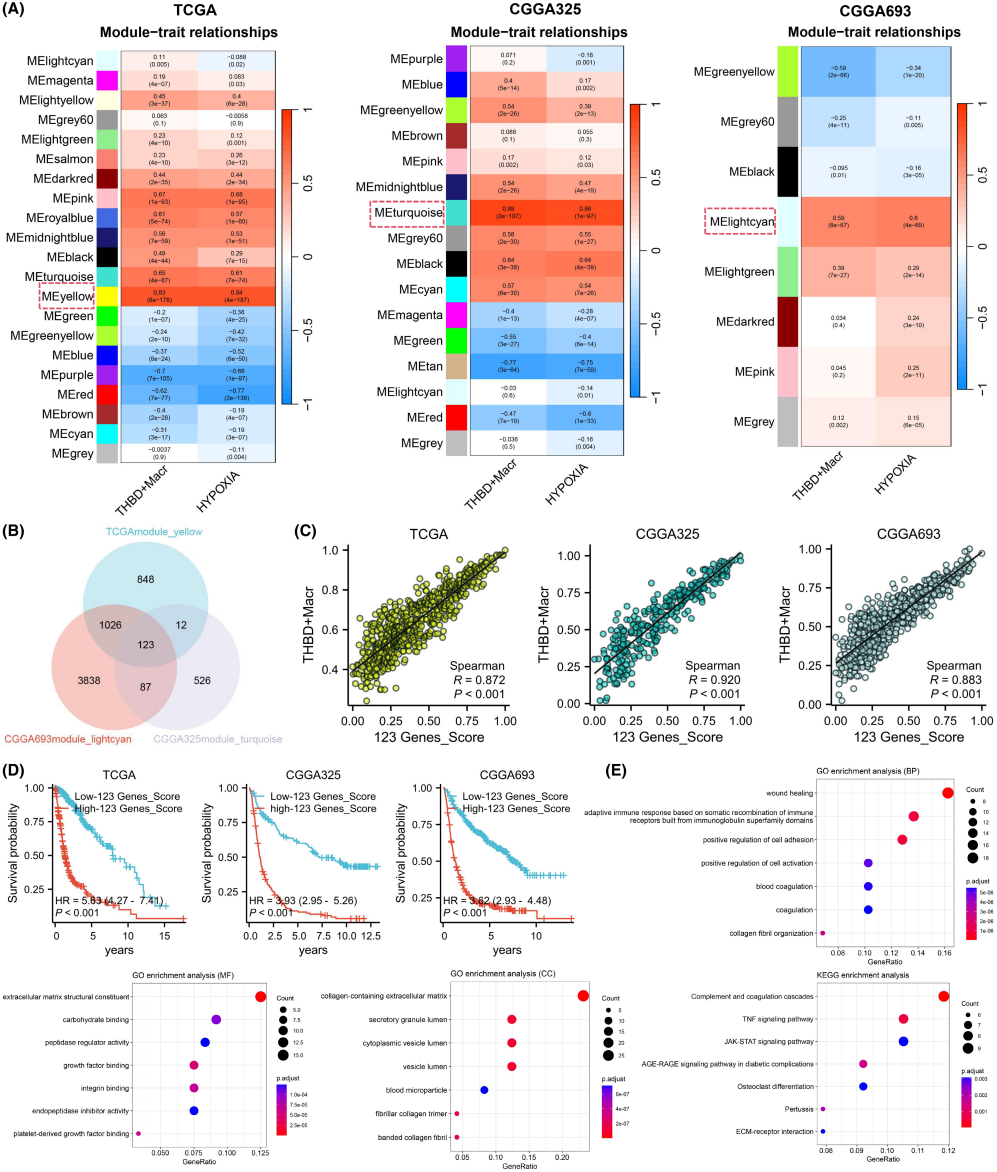
Identification of key genes associated with hypoxia in THBD^+^ macrophages. (A) Heatmap displaying key modules of genes associated with THBD^+^ macrophages and hypoxia in the TCGA, CGGA325 and CGGA693 databases. (B) Cross‐analysis of common genes highly correlated with THBD^+^ macrophages and hypoxia among the three databases. (C) Correlation between the infiltration level of THBD^+^ macrophages and the scores of 123 key genes. (D) Kaplan–Meier curve analysis of survival outcomes between high and low score groups of the 123 genes. (E) GO and KEGG pathway analysis of the 123 genes.

To investigate the key genes associated with hypoxia in THBD^+^ macrophages, the WGCNA method was employed to analyse the THBD^+^ macrophage and hypoxia scores from three datasets. Gene modules that exhibited high correlation between the two phenotypes were identified (Figure 5A; Figure S3A–C). Through module cross‐analysis across the three datasets, a total of 123 key genes were identified (Figure 5B; Table S3). Subsequent analysis indicated that the scores of these 123 key genes were positively correlated with the infiltration level of THBD^+^ macrophages, impacting the prognosis of glioma patients and influencing glioma progression through various pathways and processes (Figure 5C–E).

### 3.6 The THBD ^+^ macrophage‐related risk signature (THBDMRS) demonstrates robust prognostic capability for gliomas

**FIGURE 6:**
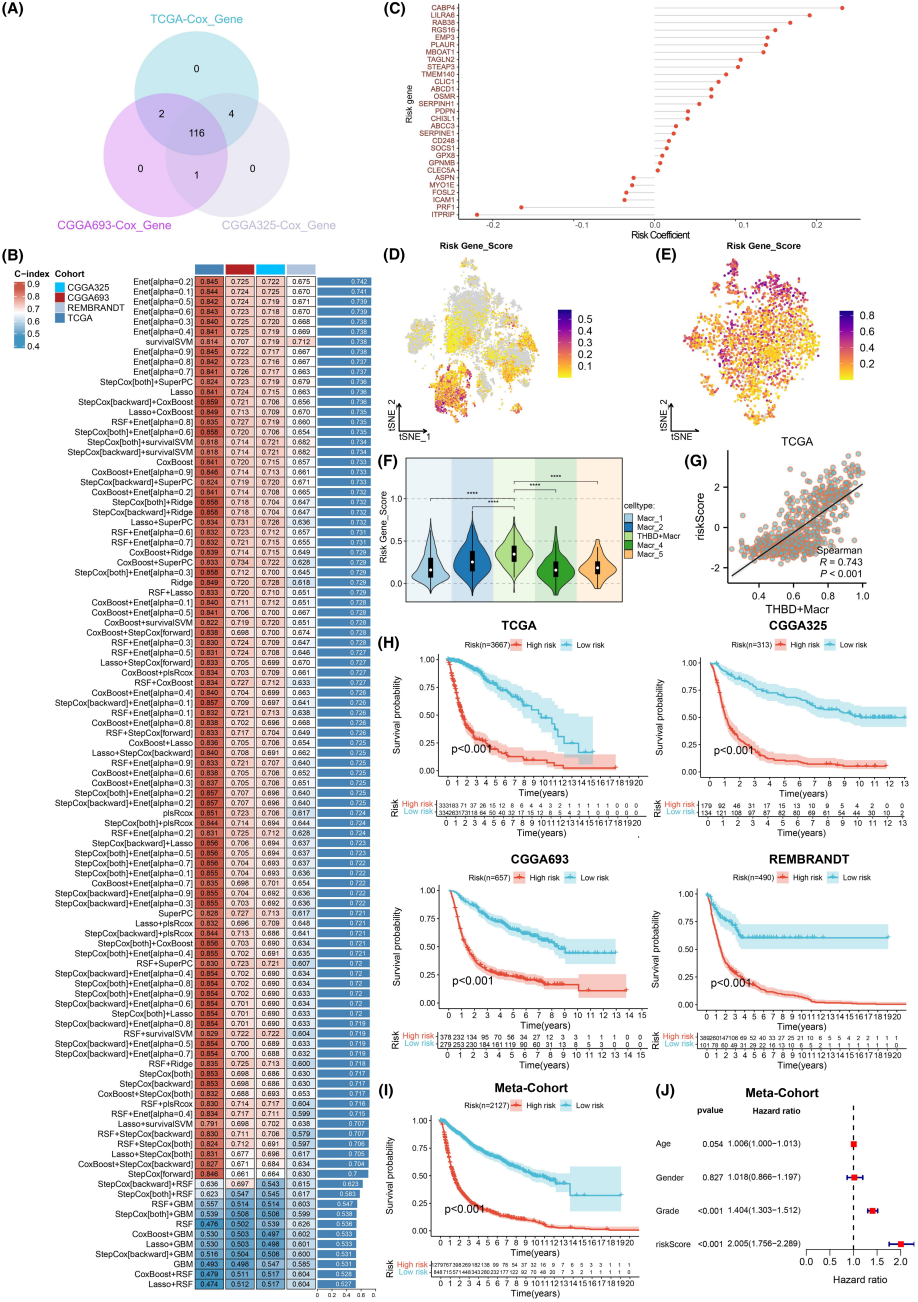
Construction of risk predictive features associated with THBD^+^ macrophages. (A) Single‐factor Cox regression analysis of the 123 genes in different databases and cross‐validation to obtain 116 commonly shared genes that are prognostically relevant. (B) Construction of 101 prediction models using the LOOCV framework, followed by calculation of the c‐index for each model across all datasets. (C) Final selection of 29 major genes using Enet [alpha = 0.2]. (D–G) t‐SNE plots showing the expression and correlation analysis of model risk scores in different immune cells and different subgroups of macrophages. (H, I) Kaplan–Meier curve analysis of survival outcomes between high and low risk score groups of the model in different databases and the overall database. (J) Multivariable Cox regression analysis of clinical records and model risk scores for all samples in the overall dataset.

Based on the 123 hypoxia‐related key genes in THBD^+^ macrophages as previously discussed, we conducted univariate Cox regression analysis across all three datasets, identifying 116 significant genes associated with prognosis through cross‐validation (Figure 6A; Table S4). Subsequently, these 116 prognostic‐related genes underwent fitting into 101 prediction models utilizing 10 distinct machine learning algorithms. The performance of these models was assessed by calculating the c‐index in the TCGA training cohort and three external validation cohorts (CGGA325, CGGA693 and REMBRABDT). Notably, the optimal model combination, Enet [alpha = 0.2], exhibited the highest average c‐index of 0.675 (Figure 6B). By applying Enet [alpha = 0.2] to the genes, we established the THBDMRS feature, comprising 29 genes, which were linked to the risk of THBD^+^ macrophages, along with corresponding risk coefficients depicted in Figure 6C. The THBDMRS risk score demonstrated a significant elevation in THBD^+^ macrophages and displayed a positive correlation with their infiltration level (Figure 6D–G; Figure S4A). Moreover, the THBDMRS risk score exhibited the highest values in gliomas, increasing with higher grades (Figure S4B). Utilizing the optimal cutoff determined by the survminer package, all patients were stratified into high‐risk and low‐risk groups. Survival analyses indicated that patients in the high THBDMRS group experienced a worse prognosis across all four datasets and even within the Meta‐Cohort (a combined cohort of the four datasets) (Figure 6H,I). Consistent findings were observed across different types and stages of glioma patients based on the Meta‐Cohort (Figure S4C). Additionally, the area under the curve (AUC) values for the 1‐, 3‐ and 5‐year OS curves, as determined by THBDMRS, confirmed its effectiveness as a predictive tool, demonstrating stability, strength and high specificity and sensitivity (Figure S5A). Consistent results were observed across various types and stages of glioma patients (Figures S4B and S5B,C). Multivariable Cox regression analysis indicated that even after adjusting for available clinical features, THBDMRS remained statistically significant, establishing its role as an independent prognostic factor for glioma patients (Figure 6J). In summary, the model exhibited strong performance in evaluating the prognosis of glioma patients.

### 3.7 Comparison of gene expression‐based prognostic signatures in gliomas

**FIGURE 7:**
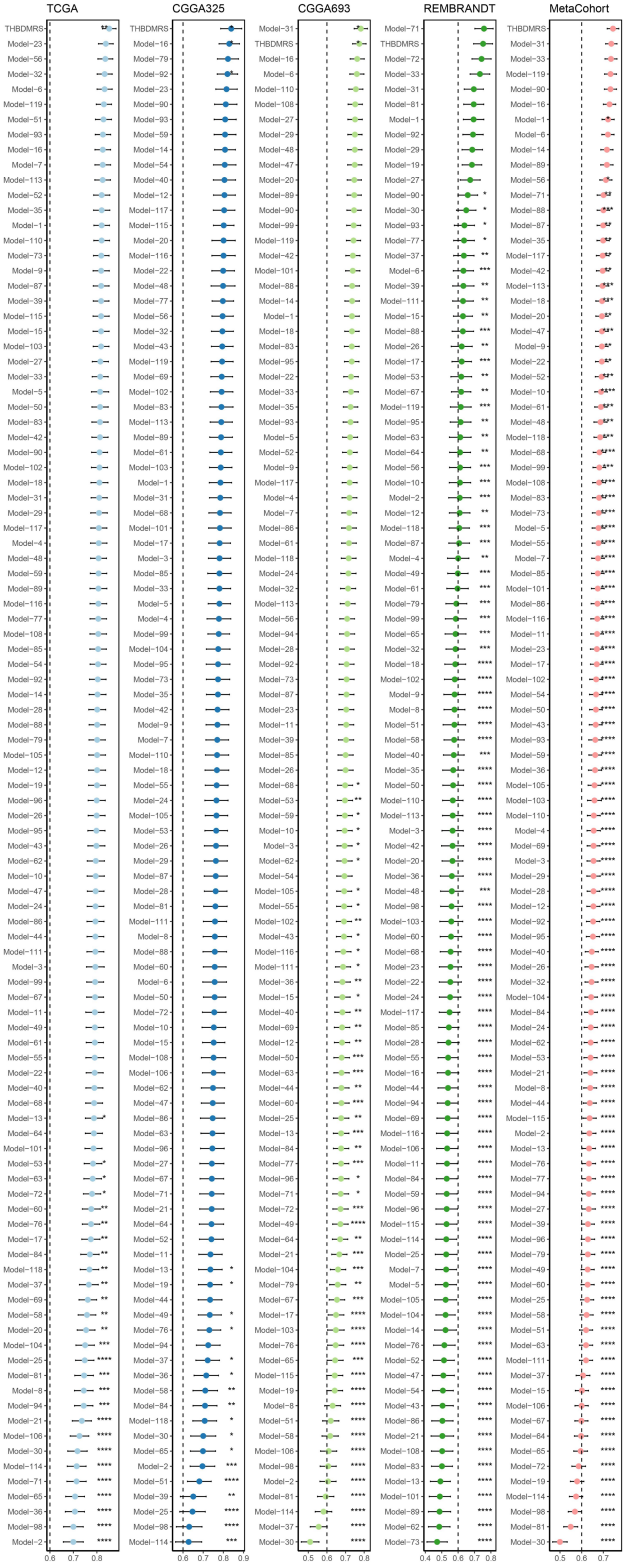
Retrieval of published signatures and comparison of the overall performance of THBDMRS with 100 other signatures.

The integration of next‐generation sequencing and big data technology has led to the development of numerous machine learning‐based prognostic and predictive gene expression signatures.^22^, ^23^ To assess the performance of THBDMRS compared to other signatures, a comprehensive search for published signatures was conducted, leading to the inclusion of 119 signatures associated with various biological processes, including autophagy, ferroptosis, epithelial‐mesenchymal transition (EMT), hypoxia, glycolysis, lipogenesis, N6‐methyladenosine and drug sensitivity (Table S2). Upon comparison, it was observed that THBDMRS outperformed all other models in the TCGA and CGGA325 datasets, securing the second position in the CGGA693 and REMBRANDT datasets. Particularly noteworthy was that THBDMRS exhibited superior performance to all other signatures in the Meta‐Cohort dataset (Figure 7). However, it was noted that while most models performed well in their respective training datasets and some external datasets, they exhibited poor performance in other datasets (Figure 7).^24^ This phenomenon could potentially be attributed to models overfitting and inadequate generalization. An interesting observation was made regarding the dimensionality reduction of the THBDMRS using two machine learning algorithms, which conferred it with enhanced potential for extrapolation to new datasets. This approach suggests that THBDMRS may offer improved generalizability and robustness across diverse datasets, representing a significant advancement in the field of prognostic and predictive gene expression signatures.

### 3.8 THBDMRS is associated with gliomas metastasis and extracellular matrix remodelling

To investigate the impact of THBDMRS on immune response, we conducted an analysis of immune infiltration‐related correlation with THBDMRS. Findings indicated a positive correlation between the THBDMRS risk score and immune score, as well as a strong association with immune cells (Figure S6A,B). Subsequently, through the selection of significantly upregulated differentially expressed genes in each dataset and cross‐analysis, we identified 295 key genes primarily linked to glioma metastasis and extracellular matrix remodelling (Figure S6C–E). Utilizing the GSVA method, we further successfully identified several key pathways influenced by THBDMRS (EPITHELIAL MESENCHYMAL_TRANSITION, angiogenesis and coagulation). These pathways exhibited significant changes in THBDMRS samples, indicating their pivotal role in the occurrence and development of THBDMRS (Figure S6F). Survival curve analysis combining THBDMRS risk score with the three pathways revealed that higher THBDMRS and pathway scores were associated with worse prognosis compared to other groups (Figure S7A–C). These results imply that THBDMRS impacts gliomas cell metastasis and extracellular matrix remodelling.

## 4 DISCUSSION

Gliomas are currently the most common malignant tumour affecting the central nervous system, significantly impacting patients' quality of life and leading to substantial losses.^25^ Studies have identified various signalling pathways and biological mechanisms in the gliomas microenvironment that contribute to its sustained occurrence and progression.^26^ Hypoxia plays a crucial role in this process, as it is recognized as a key feature of the TME, including gliomas, which contributes to the immunosuppressive milieu and the progression of gliomas.^27^ Ye et al.^28^ demonstrated that hypoxia‐inducible factor‐1α (HIF‐1α) directly binds to the HRE‐3 in the FTL promoter region to inhibit FTL expression and modulate the AKT/GSK3β/β‐catenin signalling pathway, ultimately suppressing the EMT process and reducing gliomas cell migration and invasion. Similarly, Zhang et al.^29^ reported that hypoxia induces the overexpression of circ101491 in gliomas cell‐derived exosomes, leading to the upregulation of EDN1 expression by acting as a sponge for miR‐125b‐5p, thereby promoting glioma progression. Our analysis of various databases also revealed that gliomas experiences significantly higher levels of hypoxia compared to control groups, with hypoxia levels increasing with disease progression. Gliomas patients with lower hypoxia scores exhibit better survival outcomes. Notably, the differentiation of glioma patients based on hypoxia scores can be easily achieved. These findings underscore the critical role of hypoxia in gliomas. Therefore, identifying hypoxia‐driven genes can reveal the molecular mechanisms underlying the hypoxia response in gliomas, and provide potential biomarkers for its diagnosis and therapeutic targeting.

According to reports, gliomas alter the phenotype of normal immune cells to promote its progression.^30^ This perspective was further substantiated in subsequent single‐cell sequencing, revealing that macrophages exhibited higher hypoxia scores compared to other immune cells. Moreover, as macrophage infiltration increased, their hypoxia scores correspondingly rose. Survival curve analysis also showed poor prognosis for patients with higher degrees of macrophage infiltration and high hypoxia scores. Tumour‐associated macrophages (TAMs) are the primary infiltrating immune cells in the gliomas microenvironment, facilitating gliomas progression.^31^ Hypoxia plays a crucial role in influencing TAMs' impact on tumour progression. Xu et al.^32^ observed that under hypoxic conditions, gliomas‐derived exosomes significantly promoted autophagy and M2‐like macrophage polarization, thereby facilitating gliomas proliferation and in vivo migration. Chen et al.^33^ identified that upregulated miR‐4766 in hypoxia‐treated macrophages inhibited M2 macrophage polarization by targeting VEGFA, consequently suppressing the proliferation and migration of colorectal cancer cells. These discoveries are in alignment with our own conclusions. Through temporal trajectory analysis, we determined that hypoxia drives macrophage polarization towards the M2 subtype. Additionally, we identified a pivotal hypoxia‐related gene, THBD, through innovative screening in macrophages. This gene exhibited specific upregulation in the Macr_3 macrophage subpopulation in the hypoxic core region, leading to its categorization as ‘THBD^+^ macrophages’. Subsequent investigations have demonstrated that this particular subpopulation exhibits elevated M2 polarization scores, with escalating levels of hypoxia driving macrophage polarization towards THBD^+^ macrophages. Presently, there is a paucity of literature exploring the involvement of THBD in tumours, particularly in the context of gliomas. Dong et al.^34^ identified that upregulated miR‐18a‐5p in endometrial cancer cells promoted the proliferation, migration and invasion of EC cells by targeting and downregulating THBD expression. Furthermore, abnormal methylation of THBD may be associated with gastric and colorectal cancer development.^35^ EMT is considered one of the main culprits in the metastasis and progression of glioma.^36^ Additionally, hypoxia has been shown to promote EMT.^28^ In this study, THBD^+^ macrophages were identified as a macrophage subset closely associated with hypoxia and pathways related to EMT and proliferation. We hypothesize that THBD^+^ macrophages are involved in the malignant progression of glioma. In our study, we first discovered that hypoxia could upregulate THBD expression in gliomas cells, and knocking down THBD in macrophages inhibited the proliferation, migration and invasion abilities of cocultured gliomas cells. THBD serves as a pivotal regulatory gene in THBD^+^ macrophages, making an exploration into its underlying mechanisms a crucial focus for future research. This section exclusively examined the impact of THBD on GBM cells, yet the corresponding mechanisms remain unclear. Therefore, further studies are warranted to expand the experimental scale in order to verify the relationship between THBD^+^ macrophages and the progression of glioma.

Although we have identified the critical role of hypoxia‐related THBD^+^ macrophages in gliomas cells, we are currently encountering limitations in translating these findings into tangible benefits for patient treatments. Various signatures based on multiple marker genes have been established in gliomas.^37^, ^38^, ^39^ However, these existing signatures have limitations due to overfitting. Therefore, there is an pressing need to develop a robust biomarker or gene signature for predicting patient prognosis and clinical outcomes. In our study, we conducted comprehensive multi‐parameter analysis. We performed clustering of a large number of immune cells using PCA and t‐SNE and evaluated various cell phenotypes based on hypoxia scores. Ultimately, we found that THBD^+^ macrophages in the macrophage population had the strongest correlation with hypoxia. Based on the LOOCV framework, we further selected THBD^+^ macrophage‐specific markers. Using this information, we fitted 101 combination models from 10 machine learning methods and established the THBD^+^ macrophage‐related risk signature (THBDMRS). ROC and C‐index analyses demonstrated that THBDMRS, validated in three independent datasets, showed that Enet [alpha = 0.2] was the best combination model with high accuracy and stable performance. These results indicate the considerable potential of THBDMRS for clinical applications.

Compared to other traditional clinical variables, THBDMRS emerges as an independent and superior indicator, demonstrating a notably enhanced predictive capability on prognosis as assessed by the c‐index in comparison to other factors. Moreover, we have successfully retrieved 119 previously published signatures encompassing diverse functional gene combinations. These signatures have seen limited integration into clinical practices, with even fewer undergoing validation. By comparing the predictive superiority of these features, THBDMRS also outperformed almost all models in each dataset. We observed that most models performed well in their training datasets and external datasets but showed average performance in other datasets.^24^, ^40^ This could be due to overfitting, resulting in poor generalization of the models. Our signature, achieved through dimension reduction utilizing two machine learning algorithms, exhibits enhanced potential for extrapolation.

The constructed Signature for THBDMRS comprises 29 key prognostic factors linked to hypoxia, primarily involved in EPITHELIAL_MESENCHYMAL_TRANSITION, Angiogenesis and Coagulation pathways. Many of these genes have demonstrated associations with the prognosis or progression of gliomas. Notably, EMP3, RGS16, PLAU, TAGLN2, STEAP3, CDHR1, OSMR, SERPINH1, PDPN, CHI3L1, ABCC3, SERPINE1, SOCS1, GPX8, GPNMB, CLEC5A, FOSL2, ICAM1, PRF1 and ITPRIP serve as prognostic markers for gliomas.^9^, ^41^, ^42^, ^43^, ^44^, ^45^, ^46^, ^47^, ^48^, ^49^, ^50^, ^51^, ^52^ Among them, RGS16, CLEC5A and TAGLN2 are key regulatory factors that promote glioma progression by activating the PI3K‐AKT pathway in gliomas.^53^, ^54^, ^55^, ^56^ Additionally, upregulated STEAP3 in gliomas facilitates tumour cell migration and invasion and is significantly associated with immune‐infiltrating cells, including macrophages and neutrophils, particularly M2 macrophages.^57^ Furthermore, the highly expressed CHI3L1 in gliomas enhances NF‐κB signalling by facilitating the translocation of NF‐κB subunits through binding to ACTN4 and NFKB.^58^ CHI3L1 is released into the TME, interacts with CD44 expressed on tumour‐associated macrophages, and activates the AKT pathway, thus promoting polarization of M2 macrophages. Genes like TMEM140 and GPX8 also play regulatory roles in gliomas.^59^, ^60^ These results underscore the model's pivotal predictive role in the onset and progression of gliomas.

The potential clinical translation and implementation of the THBDMRS model is enhanced by the fact that it can be replicated using a simple PCR‐based detection method, which makes it an attractive prospect for clinical applications. In clinical practice, physicians can utilize the THBDMRS score of patients to guide treatment decisions. For instance, high‐risk patients may warrant more aggressive treatment approaches, while low‐risk patients could benefit from more conservative strategies. Tailored treatment plans, encompassing surgical, radiation and chemotherapy modalities, can be devised based on individual THBDMRS scores. This approach maximizes treatment efficacy while minimizing unnecessary interventions and side effects. The THBDMRS serves as a critical tool for patient management, facilitating effective monitoring and tracking by healthcare providers. Regular assessment of THBDMRS scores enables timely adjustments to treatment plans, ensuring optimal therapeutic outcomes for patients. Despite the promising clinical significance of THBDMRS in gliomas, it's important to consider the associated limitations. One key limitation is that all samples in the study were retrospective, highlighting the need for future validation of THBDMRS in prospective multicenter cohorts. Prospective studies would enable the assessment of the model's performance in real‐time clinical scenarios, providing valuable insights into its practical utility and robustness across diverse patient populations. Additionally, it was noted that some clinical and molecular features are lacking in public datasets, which could potentially obscure the identification of associations between THBDMRS and certain variables. To address this limitation, future research efforts might benefit from the inclusion of comprehensive clinical and molecular data, enabling a more comprehensive exploration of potential associations and improving the applicability of THBDMRS in clinical practice. Therefore, extensive clinical validation and optimization of THBDMRS are necessary to ensure its accuracy and reliability in diverse patient populations. By comparing the model with actual clinical data, the predictive capability of the model can be further validated.

## 5 CONCLUSION

In summary, we have identified the most severely hypoxic subset of macrophages and constructed gliomas prognostic features based on this subset using extensive sequencing and machine learning methods. The THBDMRS model represents a promising tool that could enhance decision‐making and monitoring approaches for individual patients with gliomas.

## AUTHOR CONTRIBUTIONS

**Weichun Tang:** Data curation (equal); formal analysis (equal); methodology (equal); project administration (equal); resources (equal); software (equal); visualization (equal); writing – original draft (equal). **Juntao Du:** Data curation (equal); formal analysis (equal); methodology (equal); project administration (equal); resources (equal); software (equal); visualization (equal). **Lin Li:** Conceptualization (equal); formal analysis (equal); methodology (equal); project administration (equal); resources (equal); supervision (equal); validation (equal); writing – original draft (equal). **Shangshang Hu:** Methodology (equal); project administration (equal); software (equal). **Shuo Ma:** Investigation (equal); methodology (equal); visualization (equal). **Mengtong Xue:** methodology (equal); supervision (equal); validation (equal); writing – review and editing (equal). **Linlin Zhu:** Conceptualization (equal); funding acquisition (equal); methodology (equal); supervision (equal); validation (equal); writing – review and editing (equal).

## FUNDING INFORMATION

This project was supported by the Science Research Project of Bengbu Medical University (2023byzd121).

## CONFLICT OF INTEREST STATEMENT

The authors declare that the research was conducted in the absence of any commercial or financial relationships that could be construed as a potential conflict of interest.

## CONSENT FOR PUBLICATION

All authors have provided their consent for publication.

## Supporting information


Appendix 1.

## Data Availability

The datasets presented in this study can be found in online repositories. These can be found in the GEO database (https://www.ncbi.nlm.nih.gov/geo), China Glioma Genome Atlas (CGGA) (http://www.cgga.org.cn/), 10x Genomics website (https://www.10xgenomics.com/cn/resources/datasets/human‐glioblastoma‐wholetranscriptome‐analysis‐1‐standard‐1‐2‐0) and The Cancer Genome Atlas (TCGA) (https://portal.gdc.cancer.gov). The original contributions presented in the study are included in the article/Supplementary Material. Further inquiries can be directed to the corresponding author.
