# Supplementary material for: Hypoxia‐related THBD + macrophages as a prognostic factor in glioma: Construction of a powerful risk model
Source: J Cell Mol Med. 2024 May 29;28(10):e18393. doi: 10.1111/jcmm.18393 (PMC11135907; doi:10.1111/jcmm.18393)
Supplement: Supplementary file 1 — Appendix 1. [file JCMM-28-e18393-s001.docx]

**Supplementary material**

**Appendix 1**

**Gliomas data collection and processing**

Bulk RNA-seq datasets were obtained from the Cancer Genome Atlas (TCGA) database, respectively (https://www.cancer.gov/ccg/research/genome-sequencing/tcga)，the China Glioma Genome Atlas (CGGA) data portal (http://www.cgga.org.cn/) and the Gene Expression Omnibus (GEO) database (https://www.ncbi.nlm.nih.gov/geo/) Middle acquisition. TCGA data included glioblastoma multiforme (GBM) and brain lower grade gliomas (LGG) datasets. The CGGA database includes CGGA693 and CGGA325 datasets [1]. The GEO databases include Rembrandt (GSE108474) [2] and GSE4290 [3] datasets. Clinical information of gliomas patients included disease type, WHO grade, age, gender, and overall survival. Some samples for which clinical information was unavailable or unclear were removed, and bulk RNA-seq datasets are detailed in **supplementary Table 1**. Batch correction and integration of gene expression data were performed using the 'limma' and 'SVA' packages[4].

Single cell RNA sequencing (scRNA-seq) data were from the GSE138794 dataset. Cells within were extracted based on cell markers defined in the original study, with 19539 cells extracted from 10 gliomas patients[5]. Spatial transcriptomics data was obtained from the 10x Genomics website (https://www.10xgenomics.com/cn/resources/datasets/human-glioblastoma-wholetranscriptome-analysis-1-standard-1-2-0). The hypoxia gene set was sourced from the Molecular Signatures Database (MSigDB) (https://www.gsea-msigdb.org/gsea/msigdb), specifically from the hallmark gene sets within the database. It is worth noting that both the TCGA and GEO databases are freely accessible to the public. Therefore, this study strictly adhered to the data extraction policies of these databases, eliminating the need for ethical review and approval from an ethics committee.

**ScRNA-seq and spatial transcriptome sequencing data processing**

We conducted an analysis of single-cell RNA sequencing (scRNA-seq) data employing the R package "Seurat"[6]. To ensure the incorporation of high-quality cellular data, inclusion criteria comprised genes expressed in a minimum of three single cells, while exclusion criteria involved cells with a gene count below 200 or exceeding 10,000 reads, a count less than 1,000 reads, and a mitochondrial gene ratio exceeding 20%. Batch effects across samples were managed using the "harmony" R package. Normalization of scRNA-seq data was carried out utilizing the "Seurat" R package. Following normalization, the data were transformed into Seurat objects, and the top 2000 hypervariable genes were identified using the "FindVariableFeatures" function. Subsequently, principal component analysis (PCA) was conducted on the expression matrix of the variable genes. Clustering was performed using the "FindClusters" function, and dimensionality reduction and visualization using t-distributed stochastic neighbor embedding (t-SNE) was executed through the "RunTSNE" function. Cluster-specific markers were identified using the "FindAllMarkers" function (log2|FC| = 1, FDR = 0.05), and automatic re-annotation of all cell clusters was carried out using the "singleR" package, with annotations corrected by the CellMarker database[6, 7]. Malignant glioma cells were identified based on chromosomal copy number variations (CNVs) using the "copykat" R package[8]. Furthermore, the "ssGSEA" algorithm from the GSVA package was employed to assess the infiltration degree of cell subsets by applying it to the scRNA-seq data of this study and referencing public bulk RNA-seq data[9].

In brief, the SCTranform function was utilized to normalize the spatial transcriptome data of gliomas, followed by dimension reduction using "RunPCA" for unsupervised cluster analysis. The "FindNeighbors" and "FindClusters" functions were executed with default parameters and the 30 most significant principal components. Visualization of subpopulations and genes was achieved using the "SpatialFeaturePlot" function. Integration of surface markers or gene sets of different cell types in single-cell data enabled the adoption of the "ssGSEA" algorithm for scoring and mapping into spatial feature maps.

**Pseudotime analysis**

Pseudotime analysis were constructed using the R software package 'monocle2'[10]. After reading the UMI matrix from the single-cell Seurat object, the monocle object was created using the newCellDataSet function. The estimateSizeFactors and estimateDispersions functions were utilized for normalization and dispersion estimation. The detectGenes function was applied to filter out low-quality cells with "min_expr = 0.1". Dimensionality reduction was performed using the DDRTree method, followed by cell ordering using the orderCells function. Visualization was conducted using the 'plot_cells' function.[10].

**Gene set functional analysis**

Gene set functional analysis was conducted using the R packages "clusterProfiler" [11] and "GSVA"[9]. The analysis of specific gene sets using Gene Ontology (GO) and Kyoto Encyclopedia of Genes and Genomes (KEGG) was conducted, with a significance level set at P < 0.05 for enrichment. For Gene Set Variation Analysis (GSVA), the HALLMARK dataset from MSigDB was utilized. The GSVA matrix was clustered and visualized as a heatmap using the "Pheatmap" R package. This analysis enables exploration of functional enrichment and pathways associated with the identified gene sets, offering valuable insights into the biological processes and molecular functions underlying the observed scRNA-seq data..

**Cell–cell communication analysis**

The R package "CellChat" was utilized for performing cell-cell communication analysis. The CellChat database encompasses categories such as "secretory signaling", "ECM receptors", and "cell-cell contacts" for comprehensive analysis and interpretation of cellular communication processes. Based on ligand-receptor interaction analysis, the R software package Cellchat was employed to predict intercellular communication between GBM macrophages and malignant cells. Normalized Seurat data served as the Cellchat input, with CellChatDB.human selected as the ligand-receptor interaction database. The computeCommunProb function was utilized to calculate communication probabilities, revealing the quantity and weight of cell interactions. The netVisual_bubble function visualized the communication activity of L-R pairs between macrophages and malignant cells[12].

**Survival analysis**

In the GBM cohort, gene set enrichment analysis (GSEA) was employed to calculate the scores of gene sets in the transcriptomic data. The median score was used as the cutoff to stratify samples into high and low scoring groups. Survival analysis was then performed using the R software packages survival and survminer.

**Correlation analysis**

In order to quantify the correlation between the infiltration levels of certain cells and the degree of hypoxia or other scores, the "ssGSEA" algorithm was employed for deconvolution of bulk RNA-seq data. The Pearson correlation test was utilized to determine the correlation. A p-value of less than 0.05 and a significant correlation coefficient were considered as indicative of a significant correlation.

**Weighted correlation network analysis (WGCNA) and hdWGCNA analysis**

WGCNA is an effective technique that can decipher specific gene expression patterns involved in multiple biological processes[13]. The co-expression hypoxia gene network analysis of THBD^+^ macrophages was carried out using the WGCNA package. Initially, an appropriate soft-thresholding parameter (β) was calculated to ensure the network's adherence to the criterion of a scale-free network. Subsequently, the weighted adjacency matrix was transformed into a Topological Overlap Matrix (TOM), and the corresponding dissimilarity (1-TOM) was generated. Module identification was performed using the dynamic tree-cutting method. In order to pinpoint hypoxia gene modules significantly associated with THBD^+^ macrophages, the module exhibiting the highest correlation was selected for further investigation.

HDWGCNA is a novel algorithm that provides a highly modular approach for constructing co-expression networks across multiple scales of cellular and spatial hierarchical structures, which is more suitable for scRNA-seq data (37426759). We employed hdWGCNA to analyze our scRNA-seq data. Genes expressed in at least 5% of cells were selected to construct the hdWGCNA object, which was then transformed into Metacells object. High-dimensional weighted gene co-expression network analysis (hdWGCNA) built an unscaled network at the single-cell level using the R package "hdWGCNA." A soft threshold of 1 was chosen to achieve optimal connectivity after setting the threshold for the scale-free topology model to >0.85. A co-expression network with a soft power of 9 was constructed for subsequent analysis. Module scores were modularized for the TCGA cohort using GSVA. The correlation between modules and phenotypes was evaluated using Spearman's test. A protein-protein interaction (PPI) network was constructed using the function "HubGeneNetworkPlot." All standard downstream analyses were conducted following the official pipeline, which can be found at https://smorabit.github.io/hdWGCNA/articles/basic_tutorial.html.

**Construction and validation of the THBD^+^ macrophage risk signature (THBDMRS)**

Recognizing the significance of THBD^+^ macrophages in the context of hypoxia, a THBD^+^ macrophage associated risk signature (THBDMRS) was developed with a focus on achieving high accuracy and stable performance. This process involved the integration of 10 distinct machine learning algorithms and 101 combinations of algorithms. These included random survival forests (RSF), elastic network (eNet), Lasso, Ridge, stepwise Cox, CoxBoost, partial least squares regression for Cox (plsRcox), supported principal components (SuperPC), generalized boosted regression modeling (GBM), and survival support vector machine (survival-SVM). Through the meticulous exploration and combination of these diverse algorithms, the resulting THBDMRS aimed to provide a robust and reliable framework for assessing risk associated with THBD^+^ macrophages in hypoxic conditions.

THBDMRS was constructed as follows:

1. The WGCNA method was used to identify key hypoxia-related genes in THBD^+^ macrophages. 2. A univariate analysis was conducted to select 116 major genes associated with prognosis. 3. Using the leave-one-out cross-validation (LOOCV) framework, predictive models were built for the 116 major genes using 101 algorithm combinations. 4. All models were tested on the training cohort TCGA dataset as well as external validation cohorts CGGA325, CGGA693, and REMBRANDT datasets. 5. The concordance index (C-index) was calculated for each model in all validation datasets, and the combination model with the highest average C-index across the datasets was selected as the optimal model. Finally, a THBD^+^ Macrophage-Related Risk Score (THBDMRS) consisting of 29 key genes associated with hypoxia and prognosis in THBD^+^ macrophages were constructed. The C-index, a metric used to evaluate model performance, is commonly employed to assess the predictive accuracy of survival analysis models. The C-index ranges from 0 to 1, with values closer to 1 indicating better predictive performance[14].

The THBDMRS validation process was performed as follows:

After determining the optimal pattern, we used the median of the risk scores based on the training cohort as the threshold to classify patients in the training and validation cohorts into high- and low-risk groups. The high-risk and low-risk groups were subjected to Kaplan-Meier survival analysis and log-rank test using the "survival" and "survminer" R packages. Receiver operating characteristic (ROC) curves were created to evaluate the accuracy of this marker. Additionally, we performed multivariable Cox analysis to demonstrate the independence of this marker.

**Collection and comparison of published risk signatures**

As of November 2023, this study conducted a collection and comparison of risk signatures. A total of 119 risk signatures were identified by searching the PubMed database system using the key term "Glioma risk signatures". The study calculated the C-index of these risk signatures by matching the expression profiles and survival information of the validation set (TCGA-GBM) and training sets (CGGA325, CGGA693, and REMBRANDT). Subsequently, the prognostic value of THBDMRS was compared with these risk features.

**Total RNA extraction and quantitative real ‐ time PCR (RT ‐qPCR)**

Total RNA was extracted from GC tissues and cells using TRIzol reagent (Sigma-Aldrich, T9424, America). The extracted total RNA (1000 ng) was reverse transcribed into cDNA using a reverse transcription kit (Thermo Fisher Scientific, Waltham, MA, USA). The cDNA from tissues and cells was diluted 20-fold and 10-fold, respectively, for subsequent experiments. GADPH was used as an internal reference gene. The forward and reverse primers for THBD were 5′-TAACGAAGACACAGACTGCGATT-3′ and 5′-CTAGCCCACGAGGTCAAGGT-3′, respectively. The forward and reverse primers for GAPDH were 5′-GGAGTCCACTGGCGTCTTCA-3′ and 5′-GTCATGAGTCCTTCCACGATACC-3′, respectively. The primers were obtained from Sangon Biotech (Shanghai, China). All analyses were performed using the 2^-ΔΔCT^ method.

**Cell culture and cell transfection**

Human macrophages (THP-1) and human gliomas cells (U251MG and SW1783) were cultured in DMEM (GIBCO, c11995500bt, Canada) supplemented with 10% fetal bovine serum (FBS, GIBCO, 10091148, Canada) and 1% penicillin / streptomycin (GIBCO, 15140-122, Canada). All media were maintained at 37° C and 5% CO2. Silencing of the THBD gene was achieved using small interfering RNA (siRNA). After cells were seeded in 6-well plates, to grow to 70-80%, negative control (NC) and shTHBD were transfected to THP-1 using Lipofectamine 3000 (Invitrogen, USA). After 48 h of transfection, supernatants from each group were added to U251MG and SW1783 cells for 48 h of continuous culture, followed by in vitro functional tests of tumor cells.

**CCK-8, cloning, and Transwell experiments**

After cell transfection and co-cultivation for 48 hours, 3000 cells were evenly seeded into a 96-well plate, with 5 replicate wells for each group. After waiting for the cells to adhere to the walls based on their characteristics, 10 μL of CCK-8 reagent (Dojindo, Osaka, Japan) was added to each well. The absorbance at 450 nm and 630 nm was measured using an enzyme-linked immunosorbent assay (ELISA) reader at the same time every day for 5 consecutive days.

For the clonogenic assay, 1000 cells were evenly seeded into a 6-well plate and cultured continuously for 12 days. Afterwards, the cells were fixed, stained, and washed with PBS. Five random fields were selected under a microscope for photography and recording.

In the Transwell assay (migration and invasion assay), 500 μL of complete medium was placed in the lower chamber of a 24-well plate. Then, the Transwell insert was added, and for the invasion assay, precoated Matrigel and basal medium were prepared at a ratio of 1:6 and placed in the insert. Cell suspension, containing a predetermined number of cells, was added to the insert. After 48-72 hours, the cells were fixed, stained, and washed with PBS. Five random fields were selected under a microscope for photography and recording using a Neubauer counting chamber.

**Statistical analysis**

All data processing, statistical analysis, and plotting in the study were carried out using R software version 4.3.0. Student's t-test or Wilcoxon rank-sum test is utilized to compare continuous variables between two groups, while one-way analysis of variance or Kruskal-Wallis test is employed for differential comparisons among three groups. Pearson correlation is used to assess the correlation between normally distributed variables, whereas Spearman correlation is applied for non-normally distributed variables. The Benjamini-Hochberg (BH) method is introduced to estimate the false discovery rate of multiple tests. Kaplan-Meier analysis and log-rank tests are conducted using the "survminer" R package to evaluate survival differences among groups. Prognostic variables in different subgroup clinical characteristics were determined through the application of univariate and multivariate Cox regression analysis using the "survival" package in R. All in vitro experiments were repeated three times. A p-value less than 0.05 was considered statistically significant (*p<0.05, **p<0.01, ***p<0.001).

**Reference**

1. Zhao Z, Zhang KN, Wang Q, Li G, Zeng F, Zhang Y, Wu F, Chai R, Wang Z, Zhang C, Zhang W, Bao Z, Jiang T. Chinese Glioma Genome Atlas (CGGA): A Comprehensive Resource with Functional Genomic Data from Chinese Glioma Patients. Genomics, proteomics & bioinformatics. 2021;19(1):1-12.

2. Gusev Y, Bhuvaneshwar K, Song L, Zenklusen JC, Fine H, Madhavan S. The REMBRANDT study, a large collection of genomic data from brain cancer patients. Scientific data. 2018;5:180158.

3. Sun L, Hui AM, Su Q, Vortmeyer A, Kotliarov Y, Pastorino S, Passaniti A, Menon J, Walling J, Bailey R, Rosenblum M, Mikkelsen T, Fine HA. Neuronal and glioma-derived stem cell factor induces angiogenesis within the brain. Cancer cell. 2006;9(4):287-300.

4. Leek JT, Johnson WE, Parker HS, Jaffe AE, Storey JD. The sva package for removing batch effects and other unwanted variation in high-throughput experiments. Bioinformatics (Oxford, England). 2012;28(6):882-3.

5. Wang L, Babikir H, Müller S, Yagnik G, Shamardani K, Catalan F, Kohanbash G, Alvarado B, Di Lullo E, Kriegstein A, Shah S, Wadhwa H, Chang SM, Phillips JJ, Aghi MK, Diaz AA. The Phenotypes of Proliferating Glioblastoma Cells Reside on a Single Axis of Variation. Cancer discovery. 2019;9(12):1708-19.

6. Aran D, Looney AP, Liu L, Wu E, Fong V, Hsu A, Chak S, Naikawadi RP, Wolters PJ, Abate AR, Butte AJ, Bhattacharya M. Reference-based analysis of lung single-cell sequencing reveals a transitional profibrotic macrophage. Nature immunology. 2019;20(2):163-72.

7. Zeng X, Liao G, Li S, Liu H, Zhao X, Li S, Lei K, Zhu S, Chen Z, Zhao Y, Ren X, Su T, Cheng AS, Peng S, Lin S, Wang J, Chen S, Kuang M. Eliminating METTL1-mediated accumulation of PMN-MDSCs prevents hepatocellular carcinoma recurrence after radiofrequency ablation. Hepatology (Baltimore, Md). 2023;77(4):1122-38.

8. Gao R, Bai S, Henderson YC, Lin Y, Schalck A, Yan Y, Kumar T, Hu M, Sei E, Davis A, Wang F, Shaitelman SF, Wang JR, Chen K, Moulder S, Lai SY, Navin NE. Delineating copy number and clonal substructure in human tumors from single-cell transcriptomes. Nature biotechnology. 2021;39(5):599-608.

9. Hänzelmann S, Castelo R, Guinney J. GSVA: gene set variation analysis for microarray and RNA-seq data. BMC bioinformatics. 2013;14:7.

10. Trapnell C, Cacchiarelli D, Grimsby J, Pokharel P, Li S, Morse M, Lennon NJ, Livak KJ, Mikkelsen TS, Rinn JL. The dynamics and regulators of cell fate decisions are revealed by pseudotemporal ordering of single cells. Nature biotechnology. 2014;32(4):381-6.

11. Wu T, Hu E, Xu S, Chen M, Guo P, Dai Z, Feng T, Zhou L, Tang W, Zhan L, Fu X, Liu S, Bo X, Yu G. clusterProfiler 4.0: A universal enrichment tool for interpreting omics data. Innovation (Cambridge (Mass)). 2021;2(3):100141.

12. Jin S, Guerrero-Juarez CF, Zhang L, Chang I, Ramos R, Kuan CH, Myung P, Plikus MV, Nie Q. Inference and analysis of cell-cell communication using CellChat. Nature communications. 2021;12(1):1088.

13. Langfelder P, Horvath S. WGCNA: an R package for weighted correlation network analysis. BMC bioinformatics. 2008;9:559.

14. Poirion OB, Jing Z, Chaudhary K, Huang S, Garmire LX. DeepProg: an ensemble of deep-learning and machine-learning models for prognosis prediction using multi-omics data. Genome medicine. 2021;13(1):112.

**Supplementary Table**

**Supplementary Table 1. Detailed information on public datasets.**

| **Sample Source** | **Non-tumor(n)** | **Tumor(n)** | **survival data(n)** | **Astrocytoma** | **Glioblastoma** | **Mixed glioma** | **Oligodendroglioma** | **WHO II** | **WHO III** | **WHO IV** |
| --- | --- | --- | --- | --- | --- | --- | --- | --- | --- | --- |
| TCGA(GBM+LGG) | 5 | 676 | 670 | 194 | 161 | 131 | 190 | 0 | 0 | 0 |
| CGGA693 | 0 | 693 | 657 | 279 | 249 | 0 | 165 | 188 | 255 | 249 |
| CGGA325 | 0 | 325 | 313 | 118 | 139 | 0 | 64 | 103 | 79 | 139 |
| REMBRANDT(GSE108474) | 0 | 535 | 490 | 170 | 261 | 13 | 86 | 86 | 83 | 124 |
| GSE4290 | 23 | 157 | 0 | 0 | 0 | 0 | 0 | 0 | 0 | 0 |

**Supplementary Table 2. A total of 119 published signatures were retrieved from the literature.**

| **Model** | **ENSEMBL** | **PMID** |
| --- | --- | --- |
| Model-1 | MYL12A | 35332109 |
| Model-1 | MSN | 35332109 |
| Model-1 | S100A4 | 35332109 |
| Model-1 | CHI3L1 | 35332109 |
| Model-1 | PLAUR | 35332109 |
| Model-1 | EMP3 | 35332109 |
| Model-1 | CASP4 | 35332109 |
| Model-1 | TIMP1 | 35332109 |
| Model-1 | CCDC109B | 35332109 |
| Model-2 | CCL2 | 35985661 |
| Model-2 | CCL5 | 35985661 |
| Model-2 | CCL18? | 35985661 |
| Model-2 | CXCL16 | 35985661 |
| Model-3 | IL17RA | 36325335 |
| Model-3 | IL1R1 | 36325335 |
| Model-3 | EIF2AK3 | 36325335 |
| Model-3 | CD4 | 36325335 |
| Model-3 | PRF1 | 36325335 |
| Model-3 | CXCR3 | 36325335 |
| Model-3 | CD8A | 36325335 |
| Model-3 | BAX | 36325335 |
| Model-3 | PDIA3 | 36325335 |
| Model-3 | CASP8 | 36325335 |
| Model-3 | MYD88 | 36325335 |
| Model-3 | CASP1 | 36325335 |
| Model-4 | DEK | 35936722 |
| Model-4 | CMIP | 35936722 |
| Model-4 | OGFOD1 | 35936722 |
| Model-4 | EIF4A3 | 35936722 |
| Model-4 | CD244 | 35936722 |
| Model-4 | C1RL | 35936722 |
| Model-4 | CENPN | 35936722 |
| Model-4 | CD81 | 35936722 |
| Model-4 | ITPKC | 35936722 |
| Model-5 | U2AF1 | 36248799 |
| Model-5 | SKP1 | 36248799 |
| Model-5 | RAP1B | 36248799 |
| Model-5 | IRF9 | 36248799 |
| Model-5 | RPL39 | 36248799 |
| Model-5 | CBS | 36248799 |
| Model-5 | SH3BP5 | 36248799 |
| Model-5 | BDH1 | 36248799 |
| Model-5 | ANAPC15 | 36248799 |
| Model-5 | CCND2 | 36248799 |
| Model-5 | ATP6V0C | 36248799 |
| Model-5 | FOLR2 | 36248799 |
| Model-5 | RAPGEF3 | 36248799 |
| Model-5 | ST8SIA3 | 36248799 |
| Model-5 | PCP4 | 36248799 |
| Model-5 | MGST1 | 36248799 |
| Model-5 | CSRP1 | 36248799 |
| Model-6 | ANGPTL2 | 36291283 |
| Model-6 | BAG1 | 36291283 |
| Model-6 | CDH2 | 36291283 |
| Model-6 | IFI27 | 36291283 |
| Model-6 | PTK2B | 36291283 |
| Model-6 | SOD2 | 36291283 |
| Model-6 | UBE2C | 36291283 |
| Model-7 | UBE2D3 | 35774799 |
| Model-7 | UBE2D2 | 35774799 |
| Model-7 | USP7 | 35774799 |
| Model-7 | GRN | 35774799 |
| Model-7 | UBE2S | 35774799 |
| Model-7 | UBB | 35774799 |
| Model-7 | UBE2G2 | 35774799 |
| Model-7 | BTRC | 35774799 |
| Model-7 | CUL1 | 35774799 |
| Model-7 | USP4 | 35774799 |
| Model-7 | SIAH2 | 35774799 |
| Model-7 | UBE2Z | 35774799 |
| Model-8 | DLGAP1-AS1 | 36317420 |
| Model-8 | LINC00665 | 36317420 |
| Model-8 | MIR4500HG | 36317420 |
| Model-8 | NNT-AS1 | 36317420 |
| Model-8 | PAXIP1-AS2 | 36317420 |
| Model-8 | PITPNA-AS1 | 36317420 |
| Model-8 | SNAI3-AS1 | 36317420 |
| Model-8 | STX18-AS1 | 36317420 |
| Model-8 | TTC28-AS1 | 36317420 |
| Model-8 | UBA6-AS1 | 36317420 |
| Model-9 | NEGR1 | 36358948 |
| Model-9 | ANGPTL2 | 36358948 |
| Model-9 | TMEM100 | 36358948 |
| Model-9 | MEX3A | 36358948 |
| Model-9 | CDK4 | 36358948 |
| Model-9 | SLC2A3 | 36358948 |
| Model-9 | PBK | 36358948 |
| Model-10 | PDIA4 | 36814929 |
| Model-10 | PILRB | 36814929 |
| Model-10 | DUSP6 | 36814929 |
| Model-10 | PTPRN | 36814929 |
| Model-10 | CBLN1 | 36814929 |
| Model-11 | TUBA1C | 36474171 |
| Model-11 | RPS4X | 36474171 |
| Model-11 | KDELR2 | 36474171 |
| Model-11 | SLC40A1 | 36474171 |
| Model-12 | IRF7 | 36825022 |
| Model-12 | EN1 | 36825022 |
| Model-12 | PLOD3 | 36825022 |
| Model-12 | LOXL1 | 36825022 |
| Model-13 | CASP4 | 35547808 |
| Model-13 | CASP9 | 35547808 |
| Model-13 | GSDMC | 35547808 |
| Model-13 | IL1A | 35547808 |
| Model-14 | HOXA1 | 5677036 |
| Model-14 | KIF18A | 5677036 |
| Model-14 | FAM133A | 5677036 |
| Model-14 | HGF | 5677036 |
| Model-14 | MN1 | 5677036 |
| Model-15 | MACC1 | 36389681 |
| Model-15 | OTUD1 | 36389681 |
| Model-15 | TCHH | 36389681 |
| Model-15 | ADPRH | 36389681 |
| Model-15 | ABCG2 | 36389681 |
| Model-15 | PLBD1 | 36389681 |
| Model-15 | ANG | 36389681 |
| Model-15 | QPCT | 36389681 |
| Model-16 | ACO2 | 35903107 |
| Model-16 | PTGR1 | 35903107 |
| Model-16 | GPD1 | 35903107 |
| Model-16 | HCCS | 35903107 |
| Model-16 | ABCD1 | 35903107 |
| Model-16 | RETSAT | 35903107 |
| Model-16 | SMS | 35903107 |
| Model-16 | CA2 | 35903107 |
| Model-16 | ELOVL5 | 35903107 |
| Model-16 | SCD | 35903107 |
| Model-17 | ACOT7 | 36856519 |
| Model-17 | GALE | 36856519 |
| Model-17 | NUAK2 | 36856519 |
| Model-17 | ACTA1 | 36856519 |
| Model-17 | EEF1B22 | 36856519 |
| Model-17 | ?NMNAT3 | 36856519 |
| Model-17 | RPL39L | 36856519 |
| Model-17 | PCDHB3 | 36856519 |
| Model-17 | GUCA1A | 36856519 |
| Model-17 | MICALL2 | 36856519 |
| Model-17 | OR2F2 | 36856519 |
| Model-17 | SLC35G5 | 36856519 |
| Model-17 | MGMT | 36856519 |
| Model-17 | TSPAN4 | 36856519 |
| Model-17 | TH? | 36856519 |
| Model-17 | PDE6H | 36856519 |
| Model-17 | USP44 | 36856519 |
| Model-17 | NOL3 | 36856519 |
| Model-17 | NT5M | 36856519 |
| Model-17 | ETV4 | 36856519 |
| Model-17 | NLRP12 | 36856519 |
| Model-17 | RENBP | 36856519 |
| Model-18 | CP | 35720126 |
| Model-18 | EMP1 | 35720126 |
| Model-18 | AKR1C1 | 35720126 |
| Model-18 | FMOD | 35720126 |
| Model-18 | MYBPH | 35720126 |
| Model-18 | IFI30 | 35720126 |
| Model-18 | SRPX2 | 35720126 |
| Model-18 | PDLIM1 | 35720126 |
| Model-18 | MMP19 | 35720126 |
| Model-18 | SPOCD1 | 35720126 |
| Model-18 | FCGBP | 35720126 |
| Model-18 | NAMPT | 35720126 |
| Model-18 | SLC11A1 | 35720126 |
| Model-18 | S100A10 | 35720126 |
| Model-18 | TNC | 35720126 |
| Model-18 | CSMD3 | 35720126 |
| Model-18 | ATP1A2 | 35720126 |
| Model-18 | CUX2 | 35720126 |
| Model-18 | GALNT9 | 35720126 |
| Model-18 | TNFAIP6 | 35720126 |
| Model-18 | C15orf48 | 35720126 |
| Model-18 | WSCD2 | 35720126 |
| Model-18 | CBLN1 | 35720126 |
| Model-19 | LITAF | 35429411 |
| Model-19 | MTHFD2 | 35429411 |
| Model-19 | NRXN3 | 35429411 |
| Model-19 | OSMR | 35429411 |
| Model-19 | RUFY2 | 35429411 |
| Model-20 | CRNDE | 36060266 |
| Model-20 | HAR1A | 36060266 |
| Model-20 | FAM181A-AS1 | 36060266 |
| Model-21 | WTAP | 36203569 |
| Model-21 | TRMT6 | 36203569 |
| Model-21 | DNMT1 | 36203569 |
| Model-21 | DNMT3B | 36203569 |
| Model-22 | ISCU | 35756689 |
| Model-22 | NFS1 | 35756689 |
| Model-22 | MTOR | 35756689 |
| Model-22 | EIF2S1 | 35756689 |
| Model-22 | HSPA5 | 35756689 |
| Model-22 | AURKA | 35756689 |
| Model-22 | RPL8 | 35756689 |
| Model-23 | SP1 | 35276059 |
| Model-23 | G6PD | 35276059 |
| Model-23 | ELAVL1 | 35276059 |
| Model-23 | NNMT | 35276059 |
| Model-23 | ARNTL | 35276059 |
| Model-23 | CASP6 | 35276059 |
| Model-24 | CTC-548K16.2 | 35769464 |
| Model-24 | EFHB | 35769464 |
| Model-24 | METTL7B | 35769464 |
| Model-24 | MLLT3 | 35769464 |
| Model-24 | SEL1L3 | 35769464 |
| Model-24 | SOX13 | 35769464 |
| Model-24 | FAM66C | 35769464 |
| Model-25 | CASP5 | 35990696 |
| Model-25 | GSDMB | 35990696 |
| Model-25 | GZMA | 35990696 |
| Model-25 | GZMB | 35990696 |
| Model-25 | MEFV | 35990696 |
| Model-25 | NLRC4 | 35990696 |
| Model-25 | SARM1 | 35990696 |
| Model-25 | STAT3 | 35990696 |
| Model-26 | ZBP1 | 36186436 |
| Model-26 | PLK1 | 36186436 |
| Model-26 | CFLAR | 36186436 |
| Model-26 | SQSTM1 | 36186436 |
| Model-26 | FADD | 36186436 |
| Model-27 | RAB42 | 37698534 |
| Model-27 | SH2D4A | 37698534 |
| Model-27 | GDF15 | 37698534 |
| Model-28 | ALKBH5 | 34432221 |
| Model-28 | HNRNPA2B1 | 34432221 |
| Model-28 | IGF2BP2 | 34432221 |
| Model-28 | IGF2BP3 | 34432221 |
| Model-28 | METTL14 | 34432221 |
| Model-28 | RBM15 | 34432221 |
| Model-28 | WTAP | 34432221 |
| Model-28 | YTHDC1 | 34432221 |
| Model-28 | YTHDF1 | 34432221 |
| Model-28 | YTHDF2 | 34432221 |
| Model-28 | YTHDF3 | 34432221 |
| Model-29 | BCAT1 | 35873494 |
| Model-29 | HPX | 35873494 |
| Model-29 | NNMT | 35873494 |
| Model-29 | TBX5 | 35873494 |
| Model-29 | RAB42 | 35873494 |
| Model-29 | TNFRSF19 | 35873494 |
| Model-30 | MAP1LC3A | 35692054 |
| Model-30 | TOMM20 | 35692054 |
| Model-30 | PHB2 | 35692054 |
| Model-30 | UBA52 | 35692054 |
| Model-31 | WT1 | 36118887 |
| Model-31 | HOXA2 | 36118887 |
| Model-31 | HOXC6 | 36118887 |
| Model-31 | MMP9 | 36118887 |
| Model-31 | SHOX2 | 36118887 |
| Model-31 | MYOD1 | 36118887 |
| Model-32 | GNAI3 | 36860853 |
| Model-32 | ACACB | 36860853 |
| Model-32 | ADCY3 | 36860853 |
| Model-32 | GLB1 | 36860853 |
| Model-32 | G6PC3 | 36860853 |
| Model-32 | PAFAH2 | 36860853 |
| Model-32 | NEU4 | 36860853 |
| Model-32 | LCAT | 36860853 |
| Model-32 | HADHA | 36860853 |
| Model-32 | SGPL1 | 36860853 |
| Model-32 | ACADSB | 36860853 |
| Model-33 | S100A4 | 35574375 |
| Model-33 | PLAUR | 35574375 |
| Model-33 | EMP3 | 35574375 |
| Model-34 | TRAM2 | 34462884 |
| Model-34 | SMAGP | 34462884 |
| Model-34 | KDELC2 | 34462884 |
| Model-34 | C7ORF25 | 34462884 |
| Model-35 | POC1A | 37386136 |
| Model-35 | TMEM138 | 37386136 |
| Model-35 | TMEM237 | 37386136 |
| Model-35 | TCTN1 | 37386136 |
| Model-35 | TTC26 | 37386136 |
| Model-35 | ARL3 | 37386136 |
| Model-35 | CEP83 | 37386136 |
| Model-35 | IFT74 | 37386136 |
| Model-35 | NEK1 | 37386136 |
| Model-36 | ALDH3B1 | 35155681 |
| Model-36 | CTSZ | 35155681 |
| Model-37 | SIRT1 | 36568393 |
| Model-37 | SIRT2 | 36568393 |
| Model-37 | SIRT5 | 36568393 |
| Model-37 | SIRT6 | 36568393 |
| Model-37 | SIRT7 | 36568393 |
| Model-38 | YNX1 | 35370869 |
| Model-38 | PRELID1P4 | 35370869 |
| Model-38 | MMP9 | 35370869 |
| Model-38 | TCF12 | 35370869 |
| Model-38 | RGS14 | 35370869 |
| Model-38 | RUNX1 | 35370869 |
| Model-38 | CCR2 | 35370869 |
| Model-39 | ABCA5 | 36086728 |
| Model-39 | ABCG4 | 36086728 |
| Model-39 | ACAD11 | 36086728 |
| Model-39 | AGMO | 36086728 |
| Model-39 | ALDH1A3 | 36086728 |
| Model-39 | ALOX15B | 36086728 |
| Model-39 | APOBR | 36086728 |
| Model-39 | BMP2 | 36086728 |
| Model-39 | CRABP1 | 36086728 |
| Model-39 | CYP2E1 | 36086728 |
| Model-39 | ECHDC2 | 36086728 |
| Model-39 | EPHA8 | 36086728 |
| Model-39 | FLT3 | 36086728 |
| Model-39 | HTR2C | 36086728 |
| Model-39 | IL1A | 36086728 |
| Model-39 | PLCH2 | 36086728 |
| Model-39 | PLGLB1 | 36086728 |
| Model-39 | PNPLA5 | 36086728 |
| Model-39 | TSKU | 36086728 |
| Model-39 | TTC7B | 36086728 |
| Model-40 | EZH2 | 36389690 |
| Model-40 | LEF1 | 36389690 |
| Model-40 | CASP1 | 36389690 |
| Model-41 | HLA-DQA2 | 36159801 |
| Model-41 | HOXA3 | 36159801 |
| Model-41 | SAA2 | 36159801 |
| Model-42 | CORO1A | 35203944 |
| Model-42 | FASN | 35203944 |
| Model-42 | HSPA5 | 35203944 |
| Model-42 | IL2RG | 35203944 |
| Model-42 | LEF1 | 35203944 |
| Model-42 | MCM2 | 35203944 |
| Model-42 | NFIL3 | 35203944 |
| Model-42 | PML | 35203944 |
| Model-42 | RPL3 | 35203944 |
| Model-42 | TUBA1B | 35203944 |
| Model-43 | STAT3 | 7208656 |
| Model-43 | EGF | 7208656 |
| Model-43 | VCP | 7208656 |
| Model-43 | HSPA1A | 7208656 |
| Model-43 | HSPA1B | 7208656 |
| Model-43 | SP1 | 7208656 |
| Model-43 | TFAP2A | 7208656 |
| Model-43 | CLU | 7208656 |
| Model-43 | ERCC2 | 7208656 |
| Model-43 | PPARA | 7208656 |
| Model-43 | PON1 | 7208656 |
| Model-43 | FOXO4 | 7208656 |
| Model-43 | MAPT | 7208656 |
| Model-44 | RIPK1 | 35719998 |
| Model-44 | RIPK3 | 35719998 |
| Model-44 | FAS | 35719998 |
| Model-44 | FADD | 35719998 |
| Model-44 | FASLG | 35719998 |
| Model-44 | TLR3 | 35719998 |
| Model-44 | TNF | 35719998 |
| Model-45 | AL390755.1 | 35311131 |
| Model-45 | AL445524.1 | 35311131 |
| Model-45 | AL359643.3 | 35311131 |
| Model-45 | LINC00641 | 35311131 |
| Model-45 | AL117332.1 | 35311131 |
| Model-45 | LNCTAM34A | 35311131 |
| Model-45 | CRNDE | 35311131 |
| Model-45 | AP001486.2 | 35311131 |
| Model-45 | CARD8.AS1 | 35311131 |
| Model-46 | AL357060.1 | 37114036 |
| Model-46 | HOXA-AS3 | 37114036 |
| Model-46 | LINC01561 | 37114036 |
| Model-46 | Z95115.1 | 37114036 |
| Model-46 | AL353796.1 | 37114036 |
| Model-46 | LEF1-AS1 | 37114036 |
| Model-46 | AC005224.3 | 37114036 |
| Model-46 | TMEM220-AS1 | 37114036 |
| Model-47 | MARK1 | 37434432 |
| Model-47 | ITGA5 | 37434432 |
| Model-47 | NMD3 | 37434432 |
| Model-47 | HEY1 | 37434432 |
| Model-47 | COL6A1 | 37434432 |
| Model-47 | DKK3 | 37434432 |
| Model-47 | SERPINA5 | 37434432 |
| Model-47 | NRP1 | 37434432 |
| Model-47 | PLK2 | 37434432 |
| Model-47 | ANXA1 | 37434432 |
| Model-47 | SLIT2 | 37434432 |
| Model-47 | PDPN | 37434432 |
| Model-48 | ADM2 | 32894375 |
| Model-48 | AR | 32894375 |
| Model-48 | BMP8B | 32894375 |
| Model-48 | CLCF1 | 32894375 |
| Model-48 | F2RL1 | 32894375 |
| Model-48 | FAM19A3 | 32894375 |
| Model-48 | GLP1R | 32894375 |
| Model-48 | NTS | 32894375 |
| Model-48 | PRLHR | 32894375 |
| Model-48 | S100A3 | 32894375 |
| Model-48 | TNFRSF11B | 32894375 |
| Model-48 | TRDC | 32894375 |
| Model-48 | VAV3 | 32894375 |
| Model-49 | PTPRN | 36819551 |
| Model-49 | OSMR | 36819551 |
| Model-49 | MYD88 | 36819551 |
| Model-49 | EFEMP2 | 36819551 |
| Model-50 | LUM | 36035133 |
| Model-50 | SLCO2A1 | 36035133 |
| Model-50 | VEGFA | 36035133 |
| Model-50 | POSTN | 36035133 |
| Model-50 | FSTL1 | 36035133 |
| Model-50 | PRG2 | 36035133 |
| Model-50 | SERPINA5 | 36035133 |
| Model-50 | MSX1 | 36035133 |
| Model-50 | PDGFA | 36035133 |
| Model-50 | TIMP1 | 36035133 |
| Model-50 | SPP1 | 36035133 |
| Model-50 | KCNJ8 | 36035133 |
| Model-50 | ITGAV | 36035133 |
| Model-50 | TNFRSF21 | 36035133 |
| Model-51 | ALOX5AP | 35203024 |
| Model-51 | ARHGDIB | 35203024 |
| Model-51 | ARPC1B | 35203024 |
| Model-51 | CD163 | 35203024 |
| Model-51 | FPR1 | 35203024 |
| Model-51 | MSR1 | 35203024 |
| Model-51 | MYL6 | 35203024 |
| Model-51 | S100A8 | 35203024 |
| Model-51 | S100A9 | 35203024 |
| Model-51 | SLC16A3 | 35203024 |
| Model-51 | SRGN | 35203024 |
| Model-51 | TMBIM6 | 35203024 |
| Model-51 | TMSB10 | 35203024 |
| Model-51 | TREM2 | 35203024 |
| Model-51 | TSPO | 35203024 |
| Model-52 | SEMA4G | 35401698 |
| Model-52 | ARL3 | 35401698 |
| Model-52 | KLHL9 | 35401698 |
| Model-52 | RHBDF1 | 35401698 |
| Model-52 | EMILIN3 | 35401698 |
| Model-52 | TNFRSF11B | 35401698 |
| Model-52 | IGFBP2 | 35401698 |
| Model-52 | ABCC3 | 35401698 |
| Model-52 | EMP3 | 35401698 |
| Model-52 | RYR3 | 35401698 |
| Model-52 | ADAMTS3 | 35401698 |
| Model-52 | ARL9 | 35401698 |
| Model-52 | PTGFRN | 35401698 |
| Model-52 | IGF2BP2 | 35401698 |
| Model-52 | SSFA2 | 35401698 |
| Model-53 | CASP4 | 36861130 |
| Model-53 | CASP5 | 36861130 |
| Model-53 | CASP8 | 36861130 |
| Model-53 | GSDMD | 36861130 |
| Model-53 | NLRC4 | 36861130 |
| Model-54 | NRSN1 | 36138874 |
| Model-54 | ABCC8 | 36138874 |
| Model-54 | RTN1 | 36138874 |
| Model-54 | ADARB2 | 36138874 |
| Model-54 | PAQR6 | 36138874 |
| Model-54 | SPHKAP | 36138874 |
| Model-54 | FAM155A | 36138874 |
| Model-54 | GRIN3A | 36138874 |
| Model-54 | CACNG2 | 36138874 |
| Model-54 | AMZ1 | 36138874 |
| Model-54 | PCDH11Y | 36138874 |
| Model-54 | ELAVL4 | 36138874 |
| Model-54 | RPH3A | 36138874 |
| Model-55 | SIRT1 | 36895971 |
| Model-55 | SLC39A7 | 36895971 |
| Model-55 | HAT1 | 36895971 |
| Model-55 | MYCN | 36895971 |
| Model-55 | MYC | 36895971 |
| Model-55 | IDH1 | 36895971 |
| Model-55 | TNFRSF21 | 36895971 |
| Model-55 | TLR3 | 36895971 |
| Model-55 | FADD | 36895971 |
| Model-55 | STAT3 | 36895971 |
| Model-56 | FBXO18 | 35615996 |
| Model-56 | MMS19 | 35615996 |
| Model-56 | SMC4 | 35615996 |
| Model-56 | HEXB | 35615996 |
| Model-56 | UBQLN4 | 35615996 |
| Model-56 | VAV3 | 35615996 |
| Model-56 | E2F7 | 35615996 |
| Model-56 | EFNB1 | 35615996 |
| Model-56 | WEE1 | 35615996 |
| Model-56 | SAA1 | 35615996 |
| Model-56 | SHISA5 | 35615996 |
| Model-56 | WAC | 35615996 |
| Model-56 | PSMC2 | 35615996 |
| Model-56 | PTGFRN | 35615996 |
| Model-56 | EIF3L | 35615996 |
| Model-56 | HMGA2 | 35615996 |
| Model-57 | RI1 | 36867360 |
| Model-57 | RPS2 | 36867360 |
| Model-57 | BRCA1 | 36867360 |
| Model-57 | NXT1 | 36867360 |
| Model-57 | TRIM21 | 36867360 |
| Model-58 | BMPR1A | 37456651 |
| Model-58 | KLF10 | 37456651 |
| Model-58 | RAB31 | 37456651 |
| Model-58 | SMAD4 | 37456651 |
| Model-58 | ZFYVE9 | 37456651 |
| Model-59 | CRNDE | 37568715 |
| Model-59 | NRXN3 | 37568715 |
| Model-59 | POPDC3 | 37568715 |
| Model-59 | PTPRN | 37568715 |
| Model-59 | PTPRN2 | 37568715 |
| Model-59 | SLC46A2 | 37568715 |
| Model-59 | TIMP1 | 37568715 |
| Model-59 | TNFSF9 | 37568715 |
| Model-60 | IL18 | 35433410 |
| Model-60 | AOAH | 35433410 |
| Model-60 | GBP1 | 35433410 |
| Model-60 | GBP2 | 35433410 |
| Model-60 | GBP3 | 35433410 |
| Model-60 | CASP1 | 35433410 |
| Model-60 | HMOX1 | 35433410 |
| Model-60 | RELB | 35433410 |
| Model-60 | TP53 | 35433410 |
| Model-60 | TIGAR | 35433410 |
| Model-60 | IRAK4 | 35433410 |
| Model-61 | PCED1B-AS1 | 35847925 |
| Model-61 | HOTAIRM1 | 35847925 |
| Model-61 | HAR1A | 35847925 |
| Model-61 | LINC00928 | 35847925 |
| Model-61 | LINC00519 | 35847925 |
| Model-61 | LINC01088 | 35847925 |
| Model-62 | CD79B | 37635346 |
| Model-62 | CKAP4 | 37635346 |
| Model-62 | DUSP5 | 37635346 |
| Model-62 | MTHFD2 | 37635346 |
| Model-62 | OGFR | 37635346 |
| Model-62 | SPON2 | 37635346 |
| Model-62 | BANK1 | 37635346 |
| Model-62 | CXCL1 | 37635346 |
| Model-62 | CCL2 | 37635346 |
| Model-62 | CXCL6 | 37635346 |
| Model-62 | DRAM1 | 37635346 |
| Model-62 | LITAF | 37635346 |
| Model-63 | ITGA5 | 37096960 |
| Model-63 | ITGA9 | 37096960 |
| Model-63 | ITGAE | 37096960 |
| Model-63 | ITGB7 | 37096960 |
| Model-63 | ITGB8 | 37096960 |
| Model-64 | SAMD11 | 36035145 |
| Model-64 | ESPN | 36035145 |
| Model-64 | SNTG2 | 36035145 |
| Model-64 | MSH2 | 36035145 |
| Model-64 | WDR19 | 36035145 |
| Model-64 | CLOCK | 36035145 |
| Model-64 | TCF7 | 36035145 |
| Model-64 | ICA1 | 36035145 |
| Model-64 | FREM1 | 36035145 |
| Model-64 | RPS6 | 36035145 |
| Model-64 | MSMP | 36035145 |
| Model-64 | DDX31 | 36035145 |
| Model-64 | FOXN4 | 36035145 |
| Model-64 | ZCCHC8 | 36035145 |
| Model-64 | DACH1 | 36035145 |
| Model-64 | FLII | 36035145 |
| Model-64 | ARHGEF1 | 36035145 |
| Model-65 | FDX1 | 36579333 |
| Model-65 | DLD | 36579333 |
| Model-65 | LIAS | 36579333 |
| Model-65 | LIPT1 | 36579333 |
| Model-65 | PDHA1 | 36579333 |
| Model-66 | LINC01579 | 37547724 |
| Model-66 | AL022344.1 | 37547724 |
| Model-66 | AC025171.5 | 37547724 |
| Model-66 | LINC01116 | 37547724 |
| Model-66 | MIR155HG | 37547724 |
| Model-66 | AC131097.3 | 37547724 |
| Model-66 | LINC00906 | 37547724 |
| Model-66 | CYTOR | 37547724 |
| Model-66 | AC015540.1 | 37547724 |
| Model-66 | SLC25A21.AS1 | 37547724 |
| Model-66 | H19 | 37547724 |
| Model-66 | AL133415.1 | 37547724 |
| Model-66 | SNHG18 | 37547724 |
| Model-66 | FOXD3.AS1 | 37547724 |
| Model-66 | LINC02593 | 37547724 |
| Model-66 | AL354919.2 | 37547724 |
| Model-66 | CRNDE | 37547724 |
| Model-67 | AGO2 | 37679037 |
| Model-67 | CYFIP1 | 37679037 |
| Model-67 | DCP2 | 37679037 |
| Model-67 | EIF4E1B | 37679037 |
| Model-67 | EIF4G3 | 37679037 |
| Model-67 | GEMIN5 | 37679037 |
| Model-67 | METTL1 | 37679037 |
| Model-67 | NCBP1 | 37679037 |
| Model-67 | NUDT11 | 37679037 |
| Model-67 | NUDT16 | 37679037 |
| Model-67 | SNUPN | 37679037 |
| Model-67 | WDR4 | 37679037 |
| Model-67 | LARP1 | 37679037 |
| Model-68 | KLF6 | 37439825 |
| Model-68 | CHI3L1 | 37439825 |
| Model-68 | SERPINE1 | 37439825 |
| Model-68 | ANGPT2 | 37439825 |
| Model-68 | TGFBR1 | 37439825 |
| Model-68 | PTX3 | 37439825 |
| Model-69 | IL4I1 | 36407768 |
| Model-69 | CYP1A1 | 36407768 |
| Model-69 | OGDHL | 36407768 |
| Model-69 | ASMT | 36407768 |
| Model-70 | AC084824.4 | 36705778 |
| Model-70 | AC104117.3 | 36705778 |
| Model-70 | AC121761.2 | 36705778 |
| Model-70 | AL355974.2 | 36705778 |
| Model-70 | AL391834.1 | 36705778 |
| Model-70 | CRNDE | 36705778 |
| Model-70 | DNAJC9-AS1 | 36705778 |
| Model-70 | LINC01503 | 36705778 |
| Model-70 | LINC02328 | 36705778 |
| Model-70 | RNF219-AS1 | 36705778 |
| Model-70 | SNAI3-AS1 | 36705778 |
| Model-70 | TMEM220-AS1 | 36705778 |
| Model-71 | NUDT7 | 35614925 |
| Model-71 | NUDT11 | 35614925 |
| Model-71 | CYFIP2 | 35614925 |
| Model-72 | CASP1 | 36161280 |
| Model-72 | IL18 | 36161280 |
| Model-72 | CASP3 | 36161280 |
| Model-73 | DIRAS3 | 35923699 |
| Model-73 | CFLAR | 35923699 |
| Model-73 | BAX | 35923699 |
| Model-73 | TP53 | 35923699 |
| Model-73 | GRID2 | 35923699 |
| Model-73 | BIRC5 | 35923699 |
| Model-73 | MAPK9 | 35923699 |
| Model-73 | PTK6 | 35923699 |
| Model-73 | MYC | 35923699 |
| Model-74 | AL035446.1 | 35571034 |
| Model-74 | CDK6-AS1 | 35571034 |
| Model-74 | AL133425.1 | 35571034 |
| Model-74 | AGAP2AS | 35571034 |
| Model-74 | HOTAIR | 35571034 |
| Model-74 | HOXCAS | 35571034 |
| Model-75 | LDHA | 36698888 |
| Model-75 | LDHB | 36698888 |
| Model-75 | MRS2 | 36698888 |
| Model-75 | SL16A1 | 36698888 |
| Model-75 | SL25A12 | 36698888 |
| Model-76 | HSPB1 | 35711838 |
| Model-76 | STAT3 | 35711838 |
| Model-76 | CA9 | 35711838 |
| Model-76 | MAP1LC3A | 35711838 |
| Model-76 | MAPK1 | 35711838 |
| Model-76 | ZEB1 | 35711838 |
| Model-76 | TNFAIP3 | 35711838 |
| Model-77 | ATG5 | 34609723 |
| Model-77 | BCL2L1 | 34609723 |
| Model-77 | CASP3 | 34609723 |
| Model-77 | CASP8 | 34609723 |
| Model-77 | GAPDH | 34609723 |
| Model-78 | MIR22HG | 36033510 |
| Model-78 | AC083799.1 | 36033510 |
| Model-78 | PAXIP1.AS2 | 36033510 |
| Model-78 | C10orf55 | 36033510 |
| Model-78 | GNAS.AS1 | 36033510 |
| Model-78 | CRNDE | 36033510 |
| Model-78 | PCED1B.AS1 | 36033510 |
| Model-78 | LBX2.AS1 | 36033510 |
| Model-78 | LINC00641 | 36033510 |
| Model-79 | GABBR1 | 37488455 |
| Model-79 | CALCRL | 37488455 |
| Model-79 | EBI3 | 37488455 |
| Model-79 | BTG2 | 37488455 |
| Model-79 | SEMA4D | 37488455 |
| Model-79 | SELL | 37488455 |
| Model-80 | AGAP2-AS1 | 36032137 |
| Model-80 | CYP1B1-AS1 | 36032137 |
| Model-80 | UBXN10-AS1 | 36032137 |
| Model-80 | LINC01127 | 36032137 |
| Model-80 | RP11-84D1.2 | 36032137 |
| Model-81 | MAP3K15 | 35998839 |
| Model-81 | MAPK10 | 35998839 |
| Model-81 | CCL3 | 35998839 |
| Model-81 | CCL4 | 35998839 |
| Model-81 | ADAMTS1 | 35998839 |
| Model-82 | MUC12-AS1 | 36338998 |
| Model-82 | AL158212.3 | 36338998 |
| Model-82 | BASP1-AS1 | 36338998 |
| Model-82 | AL589843.1 | 36338998 |
| Model-82 | AC099850.3 | 36338998 |
| Model-82 | AC017104.1 | 36338998 |
| Model-82 | AL162511.1 | 36338998 |
| Model-82 | AC244453.3 | 36338998 |
| Model-82 | FAM181A-AS1 | 36338998 |
| Model-82 | AC002351.1 | 36338998 |
| Model-82 | LINC01537 | 36338998 |
| Model-83 | IGFBP2 | 35203526 |
| Model-83 | GPRASP1 | 35203526 |
| Model-83 | C1R | 35203526 |
| Model-83 | CHRM3 | 35203526 |
| Model-83 | CLSTN2 | 35203526 |
| Model-83 | NELL1 | 35203526 |
| Model-83 | SEZ6L2 | 35203526 |
| Model-83 | NMB | 35203526 |
| Model-83 | ICAM5 | 35203526 |
| Model-83 | HPCAL4 | 35203526 |
| Model-83 | SNAP91 | 35203526 |
| Model-83 | PCSK1N | 35203526 |
| Model-83 | PGBD5 | 35203526 |
| Model-83 | INA | 35203526 |
| Model-83 | UCHL1 | 35203526 |
| Model-83 | LHX6 | 35203526 |
| Model-84 | PTPRN | 35873480 |
| Model-84 | ABCC3 | 35873480 |
| Model-84 | MDK | 35873480 |
| Model-84 | NMB | 35873480 |
| Model-84 | RALYL | 35873480 |
| Model-85 | ETV4 | 37450027 |
| Model-85 | HMOX1 | 37450027 |
| Model-85 | MYC | 37450027 |
| Model-85 | NFE2L2 | 37450027 |
| Model-85 | UBE2C | 37450027 |
| Model-86 | AURKA | 35832194 |
| Model-86 | PCGF2 | 35832194 |
| Model-86 | MAP3K1 | 35832194 |
| Model-86 | TRIM34 | 35832194 |
| Model-86 | PRKN | 35832194 |
| Model-86 | TLE3 | 35832194 |
| Model-86 | TRIM17 | 35832194 |
| Model-87 | HSPB1 | 36531217 |
| Model-87 | HOXD10 | 36531217 |
| Model-87 | HOXA5 | 36531217 |
| Model-87 | SEC61G | 36531217 |
| Model-87 | H19 | 36531217 |
| Model-87 | ANXA2P2 | 36531217 |
| Model-87 | HOXC10 | 36531217 |
| Model-88 | PLAUR | 35372355 |
| Model-88 | ITGA5 | 35372355 |
| Model-88 | FMOD | 35372355 |
| Model-89 | IGF2BP3 | 37515613 |
| Model-89 | CENPF | 37515613 |
| Model-89 | HSPA6 | 37515613 |
| Model-89 | HOXA3 | 37515613 |
| Model-89 | NFE2L3 | 37515613 |
| Model-89 | HIST1H4I | 37515613 |
| Model-89 | LRIF1 | 37515613 |
| Model-89 | HIST1H3E | 37515613 |
| Model-89 | JUN | 37515613 |
| Model-89 | TOP2A | 37515613 |
| Model-89 | CEP85 | 37515613 |
| Model-89 | THEMIS2 | 37515613 |
| Model-89 | ALDH3A1 | 37515613 |
| Model-89 | FKBP5 | 37515613 |
| Model-89 | PITX1 | 37515613 |
| Model-89 | AHR | 37515613 |
| Model-89 | FANCF | 37515613 |
| Model-89 | GSPT1 | 37515613 |
| Model-90 | AKR1C3 | 36929451 |
| Model-90 | ACSL6 | 36929451 |
| Model-90 | SCD | 36929451 |
| Model-90 | AGPAT2 | 36929451 |
| Model-90 | FABP5 | 36929451 |
| Model-90 | PON2 | 36929451 |
| Model-90 | DPEP1 | 36929451 |
| Model-90 | CAV1 | 36929451 |
| Model-90 | PLEKHA4 | 36929451 |
| Model-90 | ALOX5 | 36929451 |
| Model-91 | AL133415.1 | 36226186 |
| Model-91 | LINC01426 | 36226186 |
| Model-91 | AC009227 | 36226186 |
| Model-92 | CDK1 | 36856182 |
| Model-92 | LOXL2 | 36856182 |
| Model-92 | LOXL3 | 36856182 |
| Model-92 | NFE2L2 | 36856182 |
| Model-92 | SLC31A1 | 36856182 |
| Model-92 | SUMF1 | 36856182 |
| Model-92 | FDX1 | 36856182 |
| Model-93 | DBI | 36726969 |
| Model-93 | FYN | 36726969 |
| Model-93 | IL18 | 36726969 |
| Model-93 | CDK1 | 36726969 |
| Model-93 | RPS3 | 36726969 |
| Model-93 | PDCD1LG2 | 36726969 |
| Model-93 | FADD | 36726969 |
| Model-93 | CXCL12 | 36726969 |
| Model-93 | CLIC1 | 36726969 |
| Model-93 | SLC10A7 | 36726969 |
| Model-93 | CDK2 | 36726969 |
| Model-93 | BATF | 36726969 |
| Model-93 | IGBP2 | 36726969 |
| Model-93 | LRRC32 | 36726969 |
| Model-94 | CD44 | 37650991 |
| Model-94 | TNFSF14 | 37650991 |
| Model-95 | IDH1 | 36246611 |
| Model-95 | TRIM24 | 36246611 |
| Model-95 | HMG20B | 36246611 |
| Model-95 | PCGF2 | 36246611 |
| Model-95 | CBX6 | 36246611 |
| Model-95 | SGF29 | 36246611 |
| Model-95 | RCC1 | 36246611 |
| Model-95 | RYBP | 36246611 |
| Model-95 | NAP1L1 | 36246611 |
| Model-95 | ZNF541 | 36246611 |
| Model-95 | CBX7 | 36246611 |
| Model-95 | USP49 | 36246611 |
| Model-95 | HNRNPA1 | 36246611 |
| Model-95 | LBR | 36246611 |
| Model-96 | UQCRB | 37091853 |
| Model-96 | CMC1 | 37091853 |
| Model-96 | COX20 | 37091853 |
| Model-97 | MPL | 36203434 |
| Model-97 | ATPAF | 36203434 |
| Model-97 | CHEK | 36203434 |
| Model-97 | PYGL | 36203434 |
| Model-97 | NGLY | 36203434 |
| Model-97 | SLC | 36203434 |
| Model-97 | COQ | 36203434 |
| Model-97 | TXN | 36203434 |
| Model-97 | SLC | 36203434 |
| Model-97 | USB | 36203434 |
| Model-97 | MPV | 36203434 |
| Model-97 | TET | 36203434 |
| Model-97 | RARS | 36203434 |
| Model-97 | DAG | 36203434 |
| Model-98 | CCDC151 | 37565732 |
| Model-98 | DRC1 | 37565732 |
| Model-98 | C2orf73 | 37565732 |
| Model-98 | CCDC13 | 37565732 |
| Model-98 | WDR63 | 37565732 |
| Model-99 | HNRNPC | 35281815 |
| Model-99 | IGF2BP2 | 35281815 |
| Model-99 | IGF2BP3 | 35281815 |
| Model-99 | LRPPRC | 35281815 |
| Model-99 | YTHDF2 | 35281815 |
| Model-100 | AC099792.1 | 36747529 |
| Model-100 | LINC01127 | 36747529 |
| Model-100 | AC004847.1 | 36747529 |
| Model-100 | DLEU1 | 36747529 |
| Model-100 | AC005005.3 | 36747529 |
| Model-100 | ELN-AS1 | 36747529 |
| Model-100 | LINC01503 | 36747529 |
| Model-100 | AL021368.2 | 36747529 |
| Model-100 | SOX21-AS1 | 36747529 |
| Model-100 | LINC00957 | 36747529 |
| Model-100 | AC002401.4 | 36747529 |
| Model-100 | AC126407.1 | 36747529 |
| Model-100 | ZNF571-AS1 | 36747529 |
| Model-100 | ZEB1-AS1 | 36747529 |
| Model-100 | UNC5B-AS1 | 36747529 |
| Model-100 | AL359504.1 | 36747529 |
| Model-100 | LINC01943 | 36747529 |
| Model-101 | BMP2 | 35186111 |
| Model-101 | F2R | 35186111 |
| Model-101 | FGF13 | 35186111 |
| Model-101 | PCSK1 | 35186111 |
| Model-101 | PRKCB | 35186111 |
| Model-101 | PTGER3 | 35186111 |
| Model-102 | CASP3 | 36605437 |
| Model-102 | CASP4 | 36605437 |
| Model-102 | CASP8 | 36605437 |
| Model-102 | CASP9 | 36605437 |
| Model-102 | GSDMC | 36605437 |
| Model-102 | IL18 | 36605437 |
| Model-102 | IL6 | 36605437 |
| Model-102 | PLCG1 | 36605437 |
| Model-102 | PRKACA | 36605437 |
| Model-103 | ASL | 36970537 |
| Model-103 | SLC7A7 | 36970537 |
| Model-103 | F13A1 | 36970537 |
| Model-103 | FAH | 36970537 |
| Model-103 | GLUD1 | 36970537 |
| Model-103 | OAT | 36970537 |
| Model-103 | FBLL1 | 36970537 |
| Model-103 | ALDH18A1 | 36970537 |
| Model-103 | ADHFE1 | 36970537 |
| Model-103 | FTCD | 36970537 |
| Model-104 | PRKCSH | 37483735 |
| Model-104 | DSEL | 37483735 |
| Model-104 | UGGT1 | 37483735 |
| Model-104 | SOD2 | 37483735 |
| Model-105 | CASP1 | 37153540 |
| Model-105 | BAX | 37153540 |
| Model-105 | CASP8 | 37153540 |
| Model-105 | CD8A | 37153540 |
| Model-105 | EIF2AK3 | 37153540 |
| Model-105 | IL1R1 | 37153540 |
| Model-105 | MYD88 | 37153540 |
| Model-105 | PRF1 | 37153540 |
| Model-105 | TNF | 37153540 |
| Model-106 | ARSB | 35966301 |
| Model-106 | CFLAR | 35966301 |
| Model-106 | WIPI2 | 35966301 |
| Model-106 | RB1 | 35966301 |
| Model-106 | ERN1 | 35966301 |
| Model-106 | RAB24 | 35966301 |
| Model-107 | GDNF-AS1 | 37403043 |
| Model-107 | HOXA-AS3 | 37403043 |
| Model-107 | LINC00346 | 37403043 |
| Model-107 | LINC00664 | 37403043 |
| Model-107 | LINC00665 | 37403043 |
| Model-107 | MIR155HG | 37403043 |
| Model-107 | NEAT1 | 37403043 |
| Model-107 | RHPN1-AS1 | 37403043 |
| Model-108 | PATZ1 | 36341390 |
| Model-108 | AURKA | 36341390 |
| Model-108 | CENPA | 36341390 |
| Model-108 | LIMK1 | 36341390 |
| Model-108 | TGFB1I1 | 36341390 |
| Model-108 | TLR3 | 36341390 |
| Model-109 | AL136964.1 | 35152365 |
| Model-109 | ARHGEF26-AS1 | 35152365 |
| Model-109 | PCED1B-AS1 | 35152365 |
| Model-109 | AS104072.1 | 35152365 |
| Model-109 | PRKCQ-AS1 | 35152365 |
| Model-109 | LINC00957 | 35152365 |
| Model-109 | AS125616.1 | 35152365 |
| Model-109 | PSMB8-AS1 | 35152365 |
| Model-109 | AC087741.1 | 35152365 |
| Model-110 | ABCC3 | 37334354 |
| Model-110 | HOXA4 | 37334354 |
| Model-110 | HOXC10 | 37334354 |
| Model-110 | NNMT | 37334354 |
| Model-110 | SCNN1B | 37334354 |
| Model-111 | AGK | 35280808 |
| Model-111 | ETV4 | 35280808 |
| Model-111 | PARD6A | 35280808 |
| Model-111 | PTP4A2 | 35280808 |
| Model-111 | RIOK3 | 35280808 |
| Model-111 | SIGMAR1 | 35280808 |
| Model-111 | SLC34A2 | 35280808 |
| Model-111 | SMURF1 | 35280808 |
| Model-111 | STK33 | 35280808 |
| Model-111 | TCEAL1 | 35280808 |
| Model-111 | TFPI | 35280808 |
| Model-111 | UROS | 35280808 |
| Model-112 | AC083864.2 | 37173373 |
| Model-112 | AC107294.1 | 37173373 |
| Model-112 | AL035446.1 | 37173373 |
| Model-112 | CRNDE | 37173373 |
| Model-112 | LINC02600 | 37173373 |
| Model-112 | SNAI3-AS1 | 37173373 |
| Model-113 | MTHFS | 37252189 |
| Model-113 | ALDH3B1 | 37252189 |
| Model-113 | GGT5 | 37252189 |
| Model-113 | DHFR | 37252189 |
| Model-113 | FKBP1B | 37252189 |
| Model-113 | ADH5 | 37252189 |
| Model-113 | NCF2 | 37252189 |
| Model-113 | GSTK1 | 37252189 |
| Model-113 | CASP3 | 37252189 |
| Model-113 | GPI | 37252189 |
| Model-113 | PDIA4 | 37252189 |
| Model-113 | MTHFD2 | 37252189 |
| Model-113 | TXN2 | 37252189 |
| Model-114 | C16orf5 | 35042833 |
| Model-114 | FANCA | 35042833 |
| Model-114 | SPARC | 35042833 |
| Model-114 | AKAP13 | 35042833 |
| Model-115 | SIRT1 | 35023963 |
| Model-115 | MTDH | 35023963 |
| Model-115 | HSPB1 | 35023963 |
| Model-115 | CISD2 | 35023963 |
| Model-115 | HMOX1 | 35023963 |
| Model-115 | ATG7 | 35023963 |
| Model-115 | MTOR | 35023963 |
| Model-115 | PRKAA2 | 35023963 |
| Model-115 | EIF2AK4 | 35023963 |
| Model-116 | KIF4A | 36837615 |
| Model-116 | KIF26A | 36837615 |
| Model-116 | KIF1A | 36837615 |
| Model-116 | KIF13A | 36837615 |
| Model-116 | KIF13B | 36837615 |
| Model-117 | IL4I1 | 36925967 |
| Model-117 | STAT1 | 36925967 |
| Model-117 | SLC36A4 | 36925967 |
| Model-117 | MAOB | 36925967 |
| Model-117 | AOX1 | 36925967 |
| Model-117 | ALDH2 | 36925967 |
| Model-118 | NBEAL1 | 37730879 |
| Model-118 | AEBP1 | 37730879 |
| Model-118 | TMEM176A | 37730879 |
| Model-118 | FASTK | 37730879 |
| Model-118 | CD81 | 37730879 |
| Model-119 | ABCC3 | 37662954 |
| Model-119 | ARHGAP12 | 37662954 |
| Model-119 | CABP4 | 37662954 |
| Model-119 | DUSP10 | 37662954 |
| Model-119 | GATA5 | 37662954 |
| Model-119 | IGF2BP2 | 37662954 |
| Model-119 | IGF2BP3 | 37662954 |
| Model-119 | LPIN3 | 37662954 |
| Model-119 | MAP3K1 | 37662954 |
| Model-119 | MSN | 37662954 |
| Model-119 | MT3 | 37662954 |
| Model-119 | NOL3 | 37662954 |
| Model-119 | PGM1 | 37662954 |
| Model-119 | PLAT | 37662954 |
| Model-119 | RRAGA | 37662954 |
| Model-119 | SP110 | 37662954 |
| Model-119 | TP73 | 37662954 |
| Model-119 | WEE1 | 37662954 |

**Supplementary Table 3. Cross-analysis of gene modules highly associated with THBD+ macrophages and hypoxia phenotype in three datasets, resulting in 123 key genes.**

| **Gene name** |
| --- |
| PLXND1 |
| TNFRSF12A |
| MRC2 |
| PLAUR |
| LTF |
| BIRC3 |
| CHI3L2 |
| BCL3 |
| RAB27A |
| FCGR2B |
| FOSL2 |
| PPP1R15A |
| ICAM1 |
| HSD3B7 |
| KDELR3 |
| PYGL |
| ABCD1 |
| TIMP1 |
| SRPX2 |
| ACP5 |
| AQP9 |
| SNX8 |
| SERPINE1 |
| SFRP4 |
| ASPN |
| COL1A1 |
| ABCC3 |
| FAM20A |
| CLEC2B |
| CISH |
| STEAP3 |
| FN1 |
| TNFAIP3 |
| LTBP2 |
| CD274 |
| TGFBI |
| PLAU |
| RAB38 |
| SNAI1 |
| F13A1 |
| TREM1 |
| IRF1 |
| CD93 |
| LIF |
| APOBEC3F |
| GDF15 |
| COL5A1 |
| EMILIN2 |
| CHI3L1 |
| LOXL2 |
| IL2RA |
| SPOCD1 |
| SLC43A3 |
| GPNMB |
| IL10 |
| IL1RN |
| EMILIN1 |
| C1RL |
| LUM |
| SLC16A3 |
| SECTM1 |
| EMP3 |
| HSPG2 |
| RGS16 |
| S100A8 |
| NFKBIZ |
| OSMR |
| TMEM140 |
| DOK2 |
| ITPRIP |
| SERPING1 |
| SERPINH1 |
| MPZL2 |
| MMP14 |
| MYO1E |
| TNFRSF14 |
| TAGLN2 |
| C1R |
| ZDHHC12 |
| HK3 |
| ITGA5 |
| PDPN |
| S100A9 |
| COL6A3 |
| NUAK2 |
| CDCP1 |
| ZC3H12A |
| GPX8 |
| COL1A2 |
| ANPEP |
| COL3A1 |
| ISG20 |
| MBOAT1 |
| HSPA6 |
| PPP1R3B |
| TNFRSF10D |
| PLK3 |
| CD248 |
| CLCF1 |
| CABP4 |
| FOSL1 |
| SPHK1 |
| CD163 |
| THBD |
| PRF1 |
| BACE2 |
| C1S |
| ANXA2 |
| UPP1 |
| TMEM255B |
| SOCS3 |
| SOCS1 |
| SERPINA3 |
| S100A4 |
| FAM114A1 |
| CFI |
| CLIC1 |
| ANG |
| HSPA7 |
| MIR155HG |
| LILRA6 |
| LBX2-AS1 |
| CLEC5A |

**Supplementary Table 4. Single-factor Cox regression analysis of 123 hypoxia-related key genes in THBD+ macrophages and cross-validation to obtain 116 major genes associated with prognosis.**

| **TCGA** | | | | |  | **CGGA325** | | | | |  | **CGGA693** | | | | | |
| --- | --- | --- | --- | --- | --- | --- | --- | --- | --- | --- | --- | --- | --- | --- | --- | --- | --- |
| **id** | **HR** | **HR.95L** | **HR.95H** | **pvalue** |  | **id** | **HR** | **HR.95L** | **HR.95H** | **pvalue** |  | **id** | **HR** | **HR.95L** | **HR.95H** | **pvalue** |  |
| ABCC3 | 1.052434858 | 1.043698952 | 1.061243884 | 2.89E-33 |  | ABCC3 | 1.013895954 | 1.010471348 | 1.017332167 | 1.30E-15 |  | ABCC3 | 1.013500454 | 1.010722192 | 1.016286352 | 1.02E-21 |  |
| ABCD1 | 1.242866555 | 1.196359174 | 1.291181869 | 5.49E-29 |  | ABCD1 | 1.232208513 | 1.180499675 | 1.286182328 | 1.34E-21 |  | ABCD1 | 1.016172005 | 1.004170515 | 1.028316933 | 0.008131993 |  |
| ACP5 | 1.054161796 | 1.035935125 | 1.072709155 | 3.08E-09 |  | ACP5 | 1.024291579 | 1.015712563 | 1.032943055 | 2.23E-08 |  | ANG | 1.038572671 | 1.026274276 | 1.051018445 | 4.75E-10 |  |
| ANG | 1.656372659 | 1.536568306 | 1.785518011 | 1.24E-39 |  | ANG | 1.102279766 | 1.074535747 | 1.130740123 | 7.04E-14 |  | ANPEP | 1.009230109 | 1.001813277 | 1.016701851 | 0.014632628 |  |
| ANPEP | 1.041237869 | 1.028636473 | 1.053993639 | 7.78E-11 |  | ANPEP | 1.019607254 | 1.011887608 | 1.027385793 | 5.51E-07 |  | ANXA2 | 1.001777168 | 1.001382567 | 1.002171924 | 1.02E-18 |  |
| ANXA2 | 1.031339986 | 1.027570385 | 1.035123416 | 2.75E-61 |  | ANXA2 | 1.002437185 | 1.001940218 | 1.002934399 | 6.51E-22 |  | APOBEC3F | 1.101587482 | 1.068970835 | 1.135199335 | 2.80E-10 |  |
| APOBEC3F | 2.756021866 | 2.423371791 | 3.13433397 | 7.84E-54 |  | APOBEC3F | 1.289511481 | 1.203574335 | 1.381584678 | 4.98E-13 |  | AQP9 | 1.032792389 | 1.013840443 | 1.052098608 | 0.000638763 |  |
| AQP9 | 1.061857426 | 1.045458206 | 1.078513888 | 4.09E-14 |  | AQP9 | 1.021680537 | 1.011051508 | 1.032421307 | 5.82E-05 |  | ASPN | 1.124360724 | 1.063883948 | 1.188275318 | 3.25E-05 |  |
| ASPN | 1.037051859 | 1.02502742 | 1.049217356 | 9.70E-10 |  | ASPN | 1.0205539 | 1.008989274 | 1.032251076 | 0.000466937 |  | BACE2 | 1.056344152 | 1.043865475 | 1.068972004 | 1.56E-19 |  |
| BACE2 | 1.347148561 | 1.291260517 | 1.405455538 | 3.18E-43 |  | BACE2 | 1.054002847 | 1.04306582 | 1.065054554 | 4.95E-23 |  | BCL3 | 1.021234255 | 1.015386302 | 1.027115888 | 7.44E-13 |  |
| BCL3 | 1.115950184 | 1.099928161 | 1.13220559 | 5.27E-50 |  | BCL3 | 1.022794301 | 1.015852132 | 1.029783912 | 8.81E-11 |  | BIRC3 | 1.048503753 | 1.024204871 | 1.073379118 | 7.52E-05 |  |
| BIRC3 | 1.264141734 | 1.208407793 | 1.322446225 | 2.23E-24 |  | BIRC3 | 1.033820753 | 1.021455821 | 1.046335365 | 6.03E-08 |  | C1R | 1.001016964 | 1.000676239 | 1.001357806 | 4.86E-09 |  |
| C1R | 1.007934601 | 1.00636047 | 1.009511195 | 3.74E-23 |  | C1R | 1.002148875 | 1.001594373 | 1.002703685 | 2.93E-14 |  | C1RL | 1.037095296 | 1.02893503 | 1.045320279 | 1.61E-19 |  |
| C1RL | 1.09770007 | 1.082689756 | 1.112918486 | 3.48E-40 |  | C1RL | 1.03594677 | 1.026280721 | 1.045703859 | 1.54E-13 |  | C1S | 1.003901113 | 1.002644494 | 1.005159306 | 1.11E-09 |  |
| C1S | 1.007942233 | 1.006063462 | 1.009824513 | 9.53E-17 |  | C1S | 1.005612496 | 1.004035278 | 1.007192191 | 2.78E-12 |  | CABP4 | 1.19955986 | 1.151612997 | 1.249502968 | 2.28E-18 |  |
| CABP4 | 3.241284191 | 2.757434763 | 3.810035091 | 4.07E-46 |  | CABP4 | 1.399289128 | 1.29358208 | 1.51363419 | 5.16E-17 |  | CD163 | 1.003392505 | 1.002000491 | 1.004786453 | 1.74E-06 |  |
| CD163 | 1.005942304 | 1.004404741 | 1.007482221 | 3.16E-14 |  | CD163 | 1.004001118 | 1.00280546 | 1.005198202 | 5.10E-11 |  | CD248 | 1.004221252 | 1.002383563 | 1.006062309 | 6.56E-06 |  |
| CD248 | 1.029965255 | 1.024564386 | 1.035394593 | 3.55E-28 |  | CD248 | 1.018269802 | 1.013109489 | 1.023456399 | 2.86E-12 |  | CD274 | 1.055751355 | 1.014681693 | 1.098483328 | 0.007363881 |  |
| CD274 | 1.279359894 | 1.221684 | 1.339758676 | 1.22E-25 |  | CD274 | 1.153926887 | 1.101770753 | 1.208552013 | 1.30E-09 |  | CD93 | 1.042053294 | 1.032076349 | 1.052126684 | 4.77E-17 |  |
| CD93 | 1.072936358 | 1.059942776 | 1.086089225 | 9.93E-30 |  | CD93 | 1.032821724 | 1.02468304 | 1.041025052 | 1.24E-15 |  | CDCP1 | 1.090647641 | 1.041302058 | 1.14233163 | 0.000239511 |  |
| CDCP1 | 1.346202835 | 1.284921452 | 1.410406893 | 6.89E-36 |  | CDCP1 | 1.066041358 | 1.042271829 | 1.090352962 | 2.72E-08 |  | CFI | 1.013270373 | 1.009592814 | 1.016961327 | 1.19E-12 |  |
| CFI | 1.33790815 | 1.269347175 | 1.410172294 | 2.08E-27 |  | CFI | 1.018115065 | 1.014049094 | 1.022197339 | 1.45E-18 |  | CHI3L1 | 1.00010174 | 1.000071775 | 1.000131706 | 2.83E-11 |  |
| CHI3L1 | 1.000419486 | 1.00035589 | 1.000483086 | 3.03E-38 |  | CHI3L1 | 1.000110361 | 1.000074209 | 1.000146515 | 2.19E-09 |  | CHI3L2 | 1.000106929 | 1.000047526 | 1.000166334 | 0.000418461 |  |
| CHI3L2 | 1.002175034 | 1.001670172 | 1.00268015 | 2.89E-17 |  | CHI3L2 | 1.001169628 | 1.000802568 | 1.001536823 | 4.16E-10 |  | CISH | 1.10143631 | 1.071303466 | 1.132416708 | 8.70E-12 |  |
| CISH | 1.123400793 | 1.092298857 | 1.155388321 | 4.55E-16 |  | CISH | 1.105172897 | 1.077179234 | 1.133894056 | 2.18E-14 |  | CLCF1 | 1.055867124 | 1.040391331 | 1.071573119 | 5.35E-13 |  |
| CLCF1 | 1.099376384 | 1.083097682 | 1.115899751 | 1.44E-35 |  | CLCF1 | 1.039039368 | 1.029344688 | 1.048825355 | 1.17E-15 |  | CLEC2B | 1.03628953 | 1.021422369 | 1.051373089 | 1.33E-06 |  |
| CLEC2B | 1.160558817 | 1.134789387 | 1.186913434 | 1.27E-38 |  | CLEC2B | 1.067834976 | 1.053454295 | 1.082411967 | 2.36E-21 |  | CLEC5A | 1.059553828 | 1.044119007 | 1.075216816 | 1.11E-14 |  |
| CLEC5A | 1.293929704 | 1.252460107 | 1.336772381 | 3.23E-54 |  | CLEC5A | 1.048087846 | 1.033780362 | 1.062593345 | 2.12E-11 |  | CLIC1 | 1.002596586 | 1.001865921 | 1.003327785 | 3.13E-12 |  |
| CLIC1 | 1.012123456 | 1.010761228 | 1.01348752 | 7.52E-69 |  | CLIC1 | 1.005002109 | 1.003947952 | 1.006057373 | 1.18E-20 |  | COL1A1 | 1.00059193 | 1.000197233 | 1.000986782 | 0.003285696 |  |
| COL1A1 | 1.002812599 | 1.002154033 | 1.003471598 | 5.31E-17 |  | COL1A1 | 1.000640565 | 1.000357437 | 1.000923772 | 9.20E-06 |  | COL1A2 | 1.000792119 | 1.000384555 | 1.001199849 | 0.000138962 |  |
| COL1A2 | 1.00211569 | 1.001526804 | 1.002704922 | 1.83E-12 |  | COL1A2 | 1.000538241 | 1.000301442 | 1.000775096 | 8.36E-06 |  | COL3A1 | 1.001764993 | 1.001137217 | 1.002393164 | 3.52E-08 |  |
| COL3A1 | 1.001195308 | 1.000861461 | 1.001529267 | 2.21E-12 |  | COL3A1 | 1.000868818 | 1.000499243 | 1.001238528 | 4.05E-06 |  | COL5A1 | 1.01025857 | 1.007362458 | 1.013163009 | 3.21E-12 |  |
| COL5A1 | 1.018219446 | 1.013119185 | 1.023345382 | 1.83E-12 |  | COL5A1 | 1.010451084 | 1.006817573 | 1.014097708 | 1.54E-08 |  | COL6A3 | 1.002290974 | 1.000284405 | 1.004301568 | 0.025215721 |  |
| COL6A3 | 1.011799746 | 1.008065068 | 1.015548261 | 5.05E-10 |  | COL6A3 | 1.005589624 | 1.003425069 | 1.007758849 | 3.98E-07 |  | DOK2 | 1.044177059 | 1.026009101 | 1.062666724 | 1.39E-06 |  |
| DOK2 | 1.272628575 | 1.218577223 | 1.329077435 | 1.32E-27 |  | DOK2 | 1.142653832 | 1.096132128 | 1.191149996 | 3.21E-10 |  | EMILIN1 | 1.000869746 | 1.000176011 | 1.001563962 | 0.013992621 |  |
| EMILIN1 | 1.014548873 | 1.011875845 | 1.017228962 | 7.29E-27 |  | EMILIN1 | 1.012031263 | 1.008897413 | 1.015174847 | 4.10E-14 |  | EMILIN2 | 1.015328048 | 1.010663686 | 1.020013936 | 9.48E-11 |  |
| EMILIN2 | 1.097150277 | 1.078099081 | 1.116538129 | 3.25E-25 |  | EMILIN2 | 1.05459638 | 1.042814792 | 1.066511074 | 1.79E-20 |  | EMP3 | 1.001616531 | 1.001165803 | 1.002067463 | 2.01E-12 |  |
| EMP3 | 1.020538572 | 1.018132449 | 1.022950383 | 6.22E-64 |  | EMP3 | 1.00722527 | 1.005574163 | 1.008879088 | 7.92E-18 |  | F13A1 | 1.002926371 | 1.001296513 | 1.004558881 | 0.000429359 |  |
| F13A1 | 1.008596268 | 1.006747748 | 1.010448182 | 5.95E-20 |  | F13A1 | 1.003387979 | 1.001561945 | 1.005217341 | 0.000273371 |  | FAM114A1 | 1.071508173 | 1.057931794 | 1.085258776 | 2.51E-26 |  |
| FAM114A1 | 1.138696634 | 1.119984468 | 1.157721433 | 2.87E-53 |  | FAM114A1 | 1.060054908 | 1.048664165 | 1.07156938 | 3.67E-26 |  | FAM20A | 1.011928255 | 1.006763574 | 1.01711943 | 5.57E-06 |  |
| FAM20A | 1.14663146 | 1.120020255 | 1.173874937 | 3.29E-30 |  | FAM20A | 1.032585058 | 1.023969202 | 1.04127341 | 6.35E-14 |  | FCGR2B | 1.01716452 | 1.011416299 | 1.02294541 | 3.96E-09 |  |
| FCGR2B | 1.367111161 | 1.303580917 | 1.433737561 | 5.85E-38 |  | FCGR2B | 1.011446666 | 1.006557829 | 1.016359247 | 4.14E-06 |  | FN1 | 1.000653216 | 1.000438142 | 1.000868337 | 2.62E-09 |  |
| FN1 | 1.003218862 | 1.002485228 | 1.003953032 | 7.30E-18 |  | FN1 | 1.00044899 | 1.000296167 | 1.000601837 | 8.45E-09 |  | FOSL1 | 1.024084574 | 1.012738122 | 1.035558149 | 2.83E-05 |  |
| FOSL1 | 1.053993503 | 1.042271926 | 1.065846903 | 3.08E-20 |  | FOSL1 | 1.044724527 | 1.032396567 | 1.057199696 | 5.04E-13 |  | FOSL2 | 1.017144277 | 1.012870088 | 1.021436502 | 2.53E-15 |  |
| FOSL2 | 1.030967006 | 1.021973121 | 1.040040042 | 8.99E-12 |  | FOSL2 | 1.025517546 | 1.018623351 | 1.032458403 | 2.45E-13 |  | GDF15 | 1.002330999 | 1.000611643 | 1.004053309 | 0.007860274 |  |
| GDF15 | 1.034621478 | 1.028078255 | 1.041206346 | 7.40E-26 |  | GDF15 | 1.021509668 | 1.015379878 | 1.027676463 | 4.20E-12 |  | GPNMB | 1.002999853 | 1.001795914 | 1.004205239 | 1.02E-06 |  |
| GPNMB | 1.017484892 | 1.014095304 | 1.020885809 | 2.41E-24 |  | GPNMB | 1.001442693 | 1.00070761 | 1.002178316 | 0.000119069 |  | GPX8 | 1.063917228 | 1.047376027 | 1.080719665 | 9.21E-15 |  |
| GPX8 | 1.125336338 | 1.106110007 | 1.14489686 | 4.03E-41 |  | GPX8 | 1.056715003 | 1.043129106 | 1.070477845 | 6.51E-17 |  | HK3 | 1.013810689 | 1.007183685 | 1.020481297 | 4.15E-05 |  |
| HK3 | 1.132299573 | 1.106104005 | 1.159115525 | 2.37E-25 |  | HK3 | 1.090960544 | 1.068114043 | 1.114295722 | 7.48E-16 |  | HSD3B7 | 1.052687374 | 1.036770846 | 1.068848253 | 3.96E-11 |  |
| HSD3B7 | 1.27183777 | 1.223887614 | 1.321666544 | 1.42E-34 |  | HSD3B7 | 1.140578208 | 1.109142299 | 1.172905091 | 2.85E-20 |  | HSPA6 | 1.019444688 | 1.014513112 | 1.024400237 | 7.04E-15 |  |
| HSPA7 | 1.056246394 | 1.042688 | 1.069981093 | 1.03E-16 |  | HSPA6 | 1.008430209 | 1.005258706 | 1.011611716 | 1.76E-07 |  | HSPA7 | 1.009829874 | 1.007134501 | 1.01253246 | 7.32E-13 |  |
| HSPG2 | 1.100996708 | 1.076625857 | 1.125919226 | 3.61E-17 |  | HSPA7 | 1.010999139 | 1.006952681 | 1.015061857 | 8.99E-08 |  | HSPG2 | 1.006434996 | 1.00459635 | 1.008277007 | 6.18E-12 |  |
| ICAM1 | 1.049001401 | 1.038549471 | 1.059558519 | 7.72E-21 |  | HSPG2 | 1.014373181 | 1.010034848 | 1.018730147 | 6.76E-11 |  | ICAM1 | 1.022215867 | 1.016117838 | 1.028350492 | 6.13E-13 |  |
| IL10 | 2.156621061 | 1.851081229 | 2.512593358 | 6.21E-23 |  | ICAM1 | 1.007039262 | 1.003829227 | 1.010259563 | 1.66E-05 |  | IL10 | 1.186436349 | 1.104832809 | 1.274067171 | 2.58E-06 |  |
| IL1RN | 1.108031995 | 1.081060201 | 1.13567672 | 3.38E-16 |  | IL10 | 1.072842003 | 1.025827658 | 1.122011045 | 0.0021031 |  | IL1RN | 1.039121718 | 1.023865666 | 1.054605092 | 3.67E-07 |  |
| IL2RA | 1.151883469 | 1.11963095 | 1.185065066 | 1.70E-22 |  | IL2RA | 1.035876338 | 1.016239704 | 1.055892408 | 0.000306549 |  | IL2RA | 1.110326031 | 1.064626729 | 1.157986983 | 1.06E-06 |  |
| IRF1 | 1.178872812 | 1.143280835 | 1.215572817 | 6.94E-26 |  | IRF1 | 1.017955337 | 1.012524132 | 1.023415676 | 7.03E-11 |  | IRF1 | 1.022741116 | 1.015247261 | 1.030290285 | 2.06E-09 |  |
| ISG20 | 1.390897851 | 1.314717272 | 1.471492672 | 1.65E-30 |  | ISG20 | 1.054413901 | 1.043141251 | 1.06580837 | 4.38E-22 |  | ISG20 | 1.007535179 | 1.004420031 | 1.010659988 | 2.02E-06 |  |
| ITGA5 | 1.065547077 | 1.055265568 | 1.07592876 | 1.06E-37 |  | ITGA5 | 1.016554741 | 1.012655859 | 1.020468634 | 5.55E-17 |  | ITGA5 | 1.025104339 | 1.019723603 | 1.030513467 | 2.61E-20 |  |
| ITPRIP | 1.133524253 | 1.088395223 | 1.180524506 | 1.48E-09 |  | ITPRIP | 1.109673621 | 1.079346131 | 1.140853253 | 1.83E-13 |  | ITPRIP | 1.083341631 | 1.062197392 | 1.10490677 | 1.72E-15 |  |
| KDELR3 | 1.053236197 | 1.041975513 | 1.064618577 | 3.16E-21 |  | KDELR3 | 1.07803442 | 1.054717575 | 1.101866735 | 1.64E-11 |  | KDELR3 | 1.039233052 | 1.026585991 | 1.052035919 | 7.27E-10 |  |
| LBX2-AS1 | 1.859777607 | 1.696574836 | 2.038679742 | 5.13E-40 |  | LBX2-AS1 | 1.271095382 | 1.198264131 | 1.348353363 | 1.61E-15 |  | LBX2-AS1 | 1.164039219 | 1.12201887 | 1.207633259 | 5.62E-16 |  |
| LIF | 1.050493039 | 1.040706235 | 1.060371879 | 6.04E-25 |  | LIF | 1.015312281 | 1.010692676 | 1.019953002 | 6.53E-11 |  | LIF | 1.022503673 | 1.016232877 | 1.028813164 | 1.34E-12 |  |
| LILRA6 | 1.751499707 | 1.606117536 | 1.910041549 | 7.92E-37 |  | LILRA6 | 1.111719068 | 1.076966525 | 1.147593038 | 6.32E-11 |  | LILRA6 | 1.086943914 | 1.055199006 | 1.119643845 | 3.53E-08 |  |
| LOXL2 | 1.024518144 | 1.018929116 | 1.030137829 | 4.00E-18 |  | LOXL2 | 1.019789981 | 1.014985621 | 1.024617083 | 4.17E-16 |  | LOXL2 | 1.011924486 | 1.008972138 | 1.014885473 | 1.84E-15 |  |
| LTBP2 | 1.073356681 | 1.056729317 | 1.090245671 | 6.27E-19 |  | LTBP2 | 1.04812671 | 1.028367752 | 1.068265315 | 1.29E-06 |  | LTBP2 | 1.023980192 | 1.009881018 | 1.038276208 | 0.000808325 |  |
| LTF | 1.000724976 | 1.000494664 | 1.000955342 | 6.78E-10 |  | LTF | 1.000203867 | 1.000052212 | 1.000355545 | 0.008418248 |  | LTF | 1.000248895 | 1.000108777 | 1.000389033 | 0.00049821 |  |
| LUM | 1.005641781 | 1.003982297 | 1.007304008 | 2.45E-11 |  | LUM | 1.000819942 | 1.000317792 | 1.001322343 | 0.001370164 |  | LUM | 1.003579806 | 1.001663211 | 1.005500068 | 0.00024845 |  |
| MBOAT1 | 1.439860417 | 1.357890368 | 1.526778647 | 3.52E-34 |  | MBOAT1 | 1.186490826 | 1.138842028 | 1.236133235 | 2.91E-16 |  | MBOAT1 | 1.170047614 | 1.117135161 | 1.225466235 | 2.91E-11 |  |
| MIR155HG | 2.03111503 | 1.858239243 | 2.220073804 | 6.00E-55 |  | MIR155HG | 1.437379656 | 1.304709206 | 1.583540813 | 2.09E-13 |  | MIR155HG | 1.304819092 | 1.229613677 | 1.384624208 | 1.57E-18 |  |
| MMP14 | 1.012714599 | 1.010856496 | 1.014576117 | 1.94E-41 |  | MMP14 | 1.007317246 | 1.00576916 | 1.008867714 | 1.53E-20 |  | MMP14 | 1.004349566 | 1.00333853 | 1.005361621 | 3.01E-17 |  |
| MPZL2 | 1.559199341 | 1.431658883 | 1.698101842 | 1.96E-24 |  | MPZL2 | 1.105147987 | 1.068730046 | 1.142806903 | 4.98E-09 |  | MPZL2 | 1.158729846 | 1.113738121 | 1.205539104 | 3.07E-13 |  |
| MRC2 | 1.035312491 | 1.030356626 | 1.040292193 | 1.30E-45 |  | MYO1E | 1.045739829 | 1.031298243 | 1.060383646 | 2.91E-10 |  | MRC2 | 1.004131195 | 1.003150933 | 1.005112415 | 1.31E-16 |  |
| MYO1E | 1.19611735 | 1.143110512 | 1.251582152 | 9.68E-15 |  | NFKBIZ | 1.038081927 | 1.026318542 | 1.049980141 | 1.30E-10 |  | MYO1E | 1.016298557 | 1.011362202 | 1.021259007 | 7.62E-11 |  |
| NFKBIZ | 1.284417467 | 1.234904621 | 1.335915504 | 9.66E-36 |  | NUAK2 | 1.052768147 | 1.030434989 | 1.075585343 | 2.60E-06 |  | NFKBIZ | 1.021679179 | 1.013051619 | 1.030380215 | 7.16E-07 |  |
| NUAK2 | 1.164182766 | 1.136288007 | 1.192762315 | 1.08E-34 |  | OSMR | 1.046069464 | 1.035435409 | 1.056812732 | 5.65E-18 |  | NUAK2 | 1.108897497 | 1.076725343 | 1.142030943 | 5.94E-12 |  |
| OSMR | 1.106145512 | 1.092354804 | 1.120110324 | 5.84E-56 |  | PDPN | 1.005207279 | 1.003945227 | 1.006470918 | 5.37E-16 |  | OSMR | 1.05920945 | 1.045400964 | 1.073200329 | 8.57E-18 |  |
| PDPN | 1.006524049 | 1.005553295 | 1.007495741 | 7.82E-40 |  | PLAU | 1.013202131 | 1.00904842 | 1.01737294 | 3.91E-10 |  | PDPN | 1.004678129 | 1.003659813 | 1.005697478 | 1.87E-19 |  |
| PLAU | 1.039532655 | 1.032785453 | 1.046323936 | 1.82E-31 |  | PLAUR | 1.010221675 | 1.007340815 | 1.013110773 | 2.96E-12 |  | PLAU | 1.012144793 | 1.008990334 | 1.015309115 | 3.46E-14 |  |
| PLAUR | 1.081515575 | 1.070066756 | 1.093086887 | 3.26E-47 |  | PLK3 | 1.050074486 | 1.038091373 | 1.062195925 | 7.18E-17 |  | PLAUR | 1.006542334 | 1.004327262 | 1.008762292 | 6.58E-09 |  |
| PLK3 | 1.211952912 | 1.181189477 | 1.243517564 | 1.27E-48 |  | PLXND1 | 1.012336027 | 1.009834519 | 1.014843732 | 2.66E-22 |  | PLK3 | 1.031692724 | 1.024042788 | 1.039399807 | 2.09E-16 |  |
| PLXND1 | 1.055649603 | 1.044086783 | 1.067340477 | 5.55E-22 |  | PPP1R15A | 1.021737285 | 1.016576414 | 1.026924357 | 8.56E-17 |  | PPP1R15A | 1.01269899 | 1.009429436 | 1.015979134 | 2.04E-14 |  |
| PPP1R15A | 1.037412099 | 1.029384452 | 1.04550235 | 1.92E-20 |  | PPP1R3B | 1.174839058 | 1.129578314 | 1.221913342 | 9.09E-16 |  | PPP1R3B | 1.137177199 | 1.100288359 | 1.175302793 | 2.17E-14 |  |
| PPP1R3B | 1.235281388 | 1.199166313 | 1.272484133 | 2.85E-44 |  | PRF1 | 1.042145014 | 1.025423391 | 1.059139317 | 5.67E-07 |  | PRF1 | 1.023703464 | 1.007652169 | 1.040010447 | 0.003668325 |  |
| PRF1 | 1.131105888 | 1.095292093 | 1.168090721 | 6.16E-14 |  | PYGL | 1.027138078 | 1.021015836 | 1.03329703 | 1.66E-18 |  | PYGL | 1.026379922 | 1.021412661 | 1.031371341 | 7.03E-26 |  |
| PYGL | 1.070729303 | 1.060466123 | 1.08109181 | 5.75E-44 |  | RAB27A | 1.122494323 | 1.094574607 | 1.151126198 | 2.43E-19 |  | RAB27A | 1.064333141 | 1.042409717 | 1.086717648 | 4.33E-09 |  |
| RAB27A | 1.363388238 | 1.305900296 | 1.423406896 | 3.67E-45 |  | RAB38 | 1.51141304 | 1.355483599 | 1.685279984 | 1.05E-13 |  | RAB38 | 1.404786941 | 1.269824615 | 1.554093632 | 4.25E-11 |  |
| RAB38 | 1.849628682 | 1.686461681 | 2.028582268 | 6.22E-39 |  | RGS16 | 1.03627831 | 1.028265842 | 1.044353212 | 2.29E-19 |  | RGS16 | 1.025129434 | 1.01994355 | 1.030341686 | 8.68E-22 |  |
| RGS16 | 1.029940868 | 1.024082806 | 1.03583244 | 3.78E-24 |  | S100A4 | 1.010616702 | 1.008186561 | 1.013052699 | 8.14E-18 |  | S100A4 | 1.001345671 | 1.000537659 | 1.002154335 | 0.001094556 |  |
| S100A4 | 1.010997537 | 1.009231166 | 1.012766999 | 1.50E-34 |  | S100A8 | 1.003474374 | 1.00213383 | 1.004816711 | 3.67E-07 |  | S100A8 | 1.000493197 | 1.000233679 | 1.000752782 | 0.000195143 |  |
| S100A8 | 1.009783164 | 1.007635106 | 1.0119358 | 3.23E-19 |  | S100A9 | 1.001648627 | 1.001028315 | 1.002269323 | 1.87E-07 |  | S100A9 | 1.00057914 | 1.000280267 | 1.000878102 | 0.000145607 |  |
| S100A9 | 1.003102128 | 1.002463871 | 1.003740791 | 1.46E-21 |  | SECTM1 | 1.060068159 | 1.040550264 | 1.079952157 | 7.64E-10 |  | SECTM1 | 1.027270095 | 1.015791401 | 1.038878501 | 2.69E-06 |  |
| SECTM1 | 1.130929924 | 1.107638106 | 1.154711531 | 4.73E-31 |  | SERPINA3 | 1.000632427 | 1.000450617 | 1.000814271 | 9.15E-12 |  | SERPINA3 | 1.000503239 | 1.000364146 | 1.000642352 | 1.32E-12 |  |
| SERPINA3 | 1.274116691 | 1.210834121 | 1.340706636 | 1.16E-20 |  | SERPINE1 | 1.004260911 | 1.003117261 | 1.005405865 | 2.60E-13 |  | SERPINE1 | 1.005745566 | 1.003811138 | 1.007683722 | 5.46E-09 |  |
| SERPINE1 | 1.003175775 | 1.002548778 | 1.003803163 | 2.79E-23 |  | SERPING1 | 1.002638641 | 1.00189777 | 1.003380059 | 2.81E-12 |  | SERPING1 | 1.00179738 | 1.001052699 | 1.002542614 | 2.21E-06 |  |
| SERPING1 | 1.021031554 | 1.017991387 | 1.024080801 | 1.34E-42 |  | SERPINH1 | 1.007126962 | 1.005679926 | 1.008576081 | 3.65E-22 |  | SERPINH1 | 1.00589426 | 1.004743584 | 1.007046254 | 8.01E-24 |  |
| SERPINH1 | 1.019654687 | 1.016732106 | 1.022585669 | 2.63E-40 |  | SFRP4 | 1.005471192 | 1.001318896 | 1.009640708 | 0.009760253 |  | SFRP4 | 1.00632252 | 1.003754166 | 1.008897446 | 1.34E-06 |  |
| SFRP4 | 1.011073276 | 1.007839544 | 1.014317383 | 1.61E-11 |  | SLC16A3 | 1.013128252 | 1.009887657 | 1.016379245 | 1.47E-15 |  | SLC16A3 | 1.004240118 | 1.003027032 | 1.005454671 | 6.84E-12 |  |
| SLC16A3 | 1.199230559 | 1.169830268 | 1.229369741 | 1.13E-46 |  | SLC43A3 | 1.025067894 | 1.018882139 | 1.031291205 | 1.08E-15 |  | SLC43A3 | 1.03694868 | 1.029518976 | 1.044432001 | 4.63E-23 |  |
| SLC43A3 | 1.316062335 | 1.265828397 | 1.36828979 | 1.64E-43 |  | SNAI1 | 1.175311594 | 1.122126878 | 1.231017072 | 8.09E-12 |  | SNAI1 | 1.085625745 | 1.056183044 | 1.115889207 | 4.73E-09 |  |
| SNAI1 | 1.102333401 | 1.076018866 | 1.129291469 | 2.71E-15 |  | SNX8 | 1.061781746 | 1.047315782 | 1.076447521 | 1.08E-17 |  | SNX8 | 1.008243895 | 1.002040838 | 1.014485352 | 0.009121788 |  |
| SNX8 | 1.121291288 | 1.088125489 | 1.155467973 | 7.83E-14 |  | SOCS1 | 1.077759826 | 1.049438956 | 1.106844983 | 3.55E-08 |  | SOCS1 | 1.082230106 | 1.06146263 | 1.103403897 | 1.31E-15 |  |
| SOCS1 | 1.236250581 | 1.196172022 | 1.277672 | 1.79E-36 |  | SOCS3 | 1.007425612 | 1.005234283 | 1.009621719 | 2.76E-11 |  | SOCS3 | 1.008069446 | 1.00620628 | 1.009936061 | 1.67E-17 |  |
| SOCS3 | 1.017566208 | 1.01493488 | 1.020204359 | 1.12E-39 |  | SPHK1 | 1.025434673 | 1.018053944 | 1.03286891 | 9.44E-12 |  | SPHK1 | 1.006651597 | 1.002701452 | 1.010617304 | 0.000950413 |  |
| SPHK1 | 1.051078084 | 1.030585011 | 1.071978659 | 7.09E-07 |  | SPOCD1 | 1.007581647 | 1.005649738 | 1.009517268 | 1.22E-14 |  | SPOCD1 | 1.003270065 | 1.002255564 | 1.004285592 | 2.54E-10 |  |
| SPOCD1 | 1.024694252 | 1.018995127 | 1.030425251 | 1.01E-17 |  | SRPX2 | 1.016343523 | 1.011174962 | 1.021538503 | 4.60E-10 |  | SRPX2 | 1.017199604 | 1.01056549 | 1.023877269 | 3.25E-07 |  |
| SRPX2 | 1.103829436 | 1.087766185 | 1.120129896 | 7.91E-40 |  | STEAP3 | 1.020384966 | 1.014446283 | 1.026358414 | 1.24E-11 |  | STEAP3 | 1.021587874 | 1.016993147 | 1.02620336 | 1.60E-20 |  |
| STEAP3 | 1.050608698 | 1.044393116 | 1.056861272 | 8.77E-60 |  | TAGLN2 | 1.003370038 | 1.002753833 | 1.003986621 | 7.06E-27 |  | TAGLN2 | 1.003022052 | 1.002515915 | 1.003528446 | 1.04E-31 |  |
| TAGLN2 | 1.006970068 | 1.006147189 | 1.00779362 | 2.90E-62 |  | TGFBI | 1.002902962 | 1.002061811 | 1.003744819 | 1.28E-11 |  | TGFBI | 1.000632251 | 1.00024127 | 1.001023385 | 0.001525328 |  |
| TGFBI | 1.009088687 | 1.007149465 | 1.011031643 | 3.01E-20 |  | THBD | 1.043492699 | 1.027514349 | 1.05971952 | 6.39E-08 |  | THBD | 1.039057017 | 1.025003373 | 1.053303348 | 3.50E-08 |  |
| THBD | 1.050303723 | 1.035016098 | 1.065817152 | 5.36E-11 |  | TIMP1 | 1.000197201 | 1.000133957 | 1.00026045 | 9.85E-10 |  | TMEM140 | 1.030845371 | 1.021801859 | 1.039968924 | 1.41E-11 |  |
| TIMP1 | 1.001561419 | 1.001350299 | 1.001772584 | 1.12E-47 |  | TMEM140 | 1.066532429 | 1.050512885 | 1.08279626 | 7.32E-17 |  | TMEM255B | 1.018206584 | 1.00985565 | 1.026626575 | 1.75E-05 |  |
| TMEM140 | 1.111101872 | 1.092509943 | 1.130010192 | 1.98E-34 |  | TMEM255B | 1.156879809 | 1.118791574 | 1.196264723 | 1.44E-17 |  | TNFAIP3 | 1.031800049 | 1.022867878 | 1.04081022 | 1.70E-12 |  |
| TMEM255B | 2.422961882 | 2.10226802 | 2.792576506 | 2.51E-34 |  | TNFAIP3 | 1.036027646 | 1.025349048 | 1.046817457 | 2.15E-11 |  | TNFRSF10D | 1.144940442 | 1.070536469 | 1.224515609 | 7.88E-05 |  |
| TNFAIP3 | 1.055591646 | 1.038692227 | 1.072766017 | 5.02E-11 |  | TNFRSF10D | 1.145567733 | 1.092866602 | 1.200810261 | 1.55E-08 |  | TNFRSF12A | 1.002847805 | 1.002161017 | 1.003535063 | 4.09E-16 |  |
| TNFRSF10D | 1.126065159 | 1.086750305 | 1.166802287 | 5.83E-11 |  | TNFRSF12A | 1.010835974 | 1.008746788 | 1.012929488 | 1.79E-24 |  | TNFRSF14 | 1.008741645 | 1.005098253 | 1.012398244 | 2.42E-06 |  |
| TNFRSF12A | 1.021657295 | 1.018980035 | 1.024341588 | 1.19E-57 |  | TNFRSF14 | 1.04003146 | 1.030797081 | 1.049348565 | 6.36E-18 |  | TREM1 | 1.004992399 | 1.002350853 | 1.007640906 | 0.000208417 |  |
| TNFRSF14 | 1.338226467 | 1.288799914 | 1.38954857 | 5.32E-52 |  | TREM1 | 1.00713612 | 1.004237982 | 1.010042621 | 1.32E-06 |  | ZC3H12A | 1.050285683 | 1.034427979 | 1.066386484 | 2.60E-10 |  |
| TREM1 | 1.099886393 | 1.080697147 | 1.119416369 | 2.91E-26 |  | UPP1 | 1.01305177 | 1.010034376 | 1.016078179 | 1.59E-17 |  | ZDHHC12 | 1.023383782 | 1.015784787 | 1.031039625 | 1.21E-09 |  |
| UPP1 | 1.082245361 | 1.070592281 | 1.094025282 | 1.91E-46 |  | ZC3H12A | 1.048276205 | 1.028673082 | 1.068252898 | 9.83E-07 |  |  |  |  |  |  |  |
| ZC3H12A | 1.196936409 | 1.153641145 | 1.241856511 | 1.14E-21 |  | ZDHHC12 | 1.064067014 | 1.050056182 | 1.078264791 | 4.21E-20 |  |  |  |  |  |  |  |
| ZDHHC12 | 1.24584191 | 1.210749716 | 1.281951212 | 2.24E-51 |  |  |  |  |  |  |  |  |  |  |  |  |  |

**Supplementary Figure**


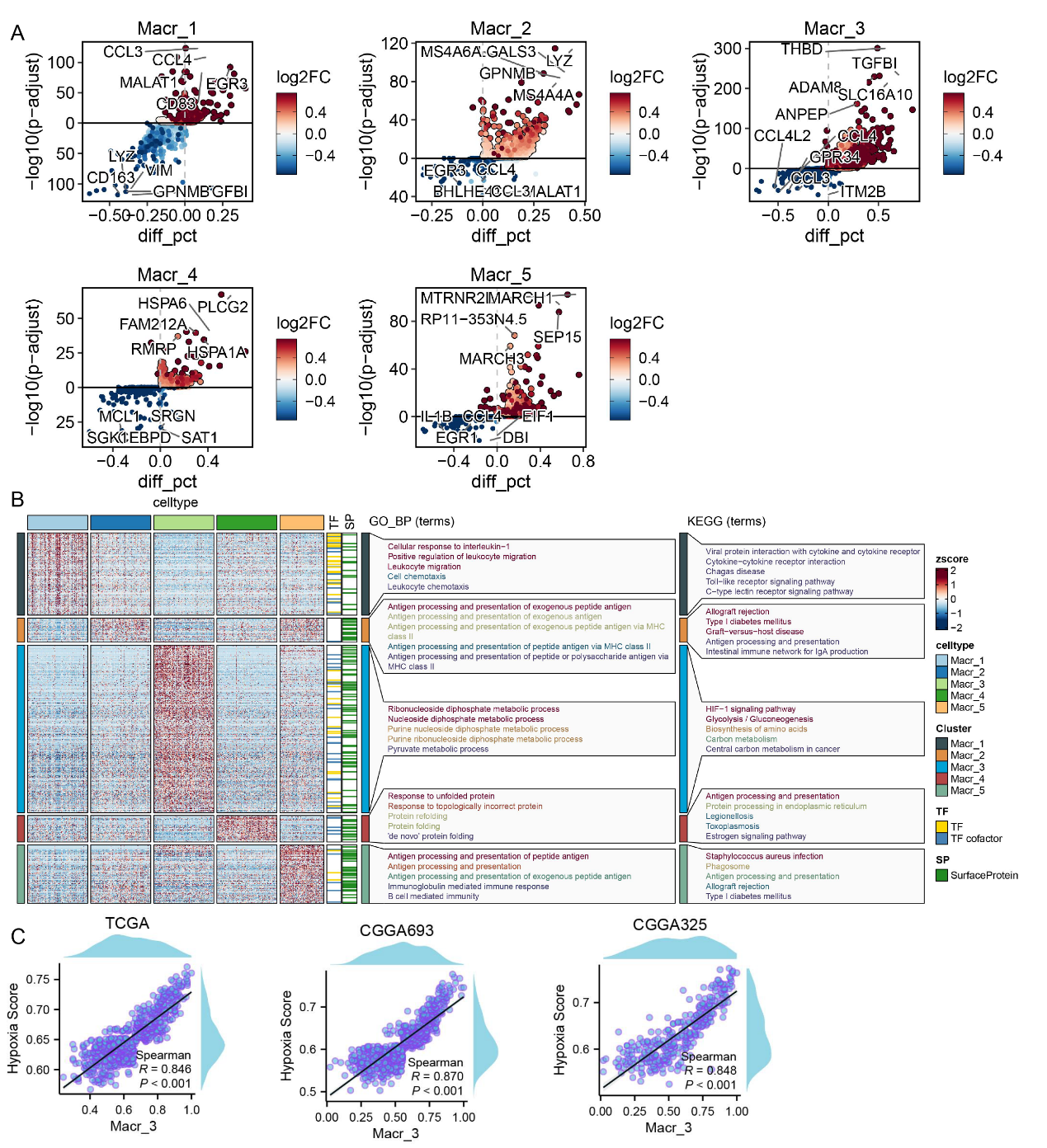


**Supplementary Figure 1. Macr_3 macrophage subset exhibits the strongest correlation with hypoxia.**

**A.** Major molecular markers of the 5 macrophage subtypes. **B.** GO and KEGG analyses of the 5 macrophage subtypes. **C.** Correlation analysis between Macr_3 macrophage subset and hypoxia score in TCGA, CGGA693, and CGGA325 databases.


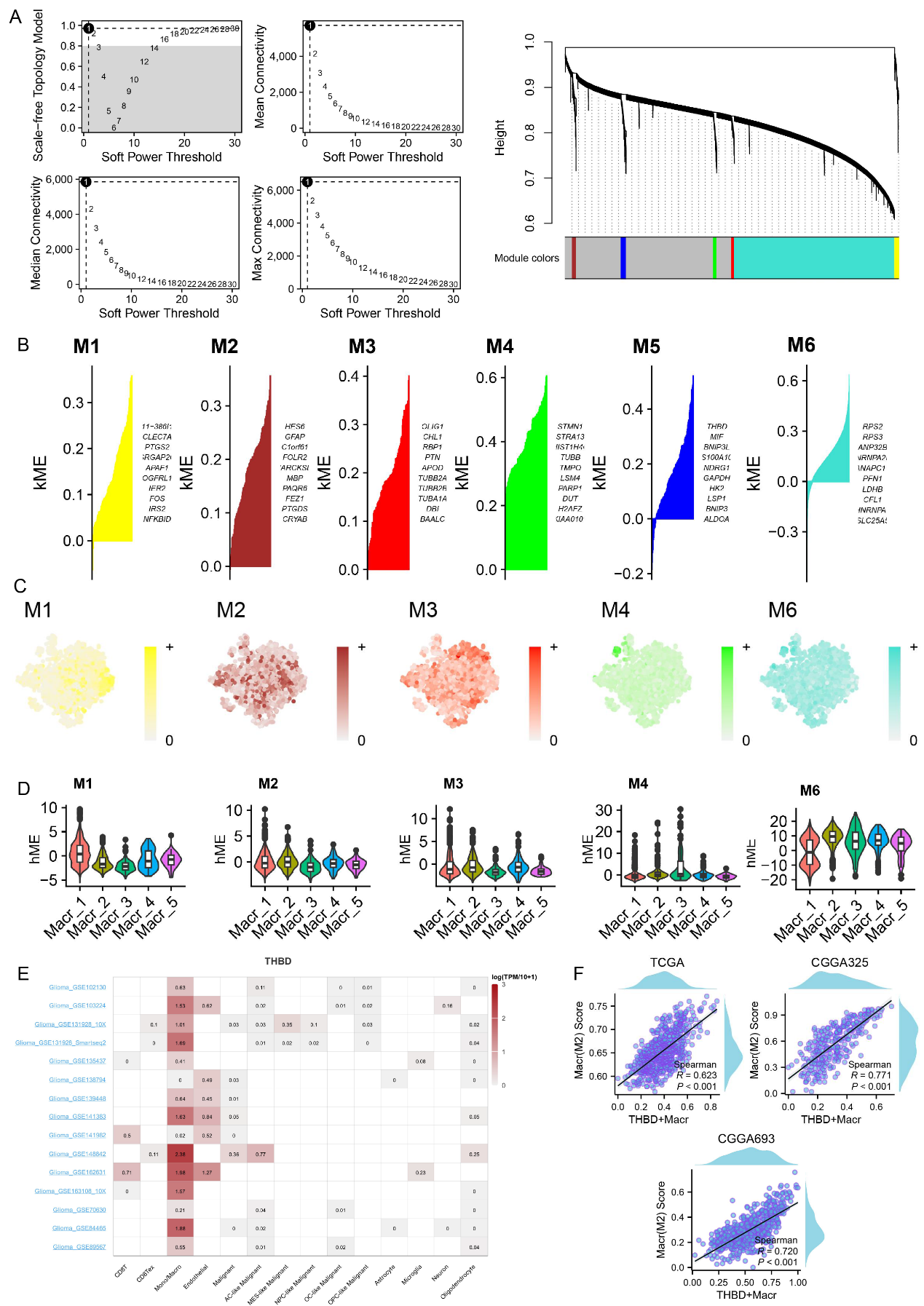


**Supplementary Figure 2. Identification of macrophage gene co-expression modules.**

**A.** Construction of weighted gene co-expression networks analysis among macrophages. **B.** Top 10 feature genes of each module ranked by feature gene connectivity (kME). **C and D.** GSVA scores of key genes in each module and comparison among macrophage subtypes. **E.** Heatmap showing the correlation of THBD molecule with various immune cells in other databases. **F.** Correlation analysis between THBD^+^ macrophages and M2 polarization score in TCGA, CGGA693, and CGGA325 databases.


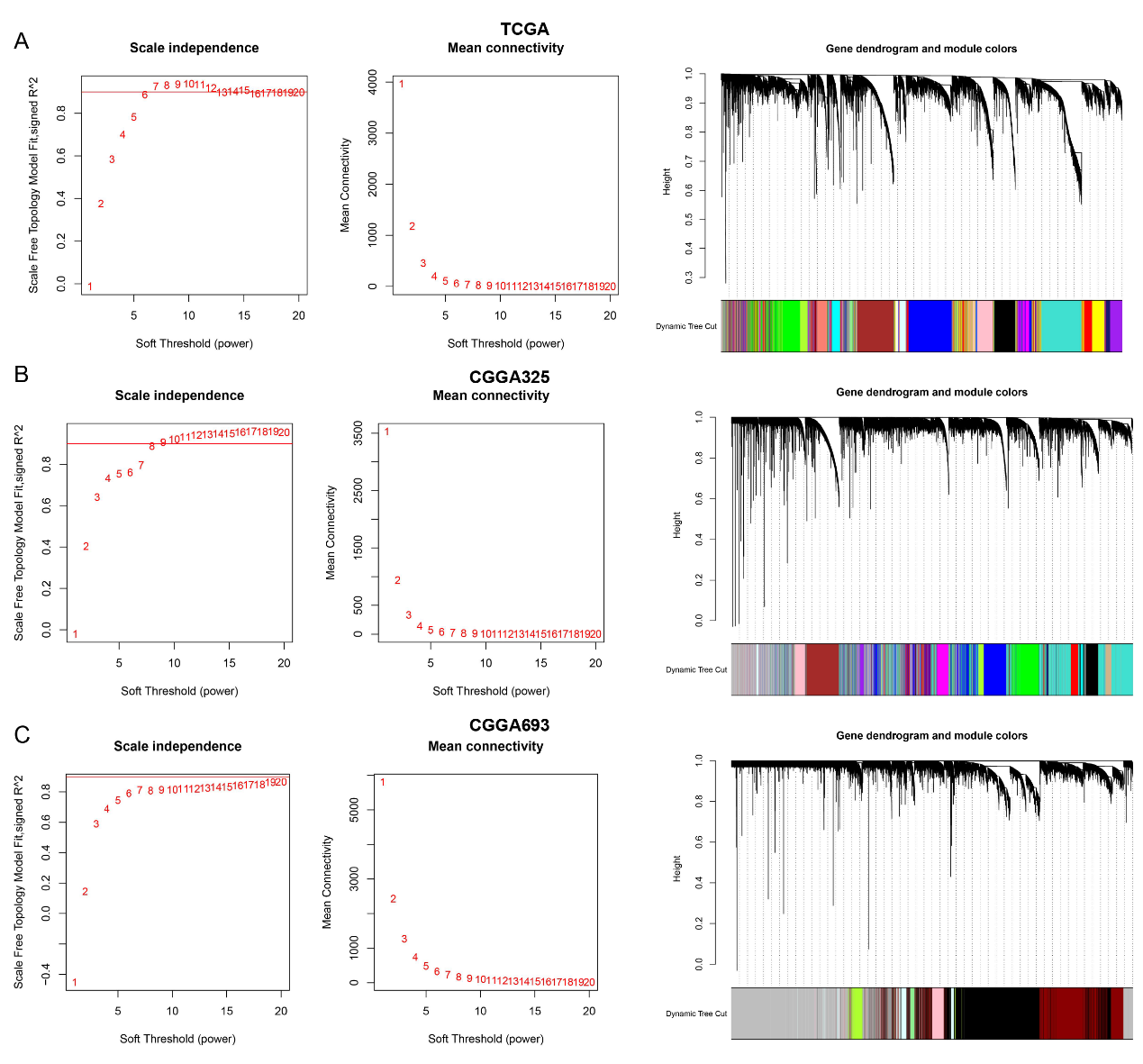


**Supplementary Figure 3. Selection of gene modules highly associated with THBD+ macrophages and hypoxia-related genes using WGCNA method in TCGA (A), CGGA325 (B), and CGGA693 (C) datasets.**


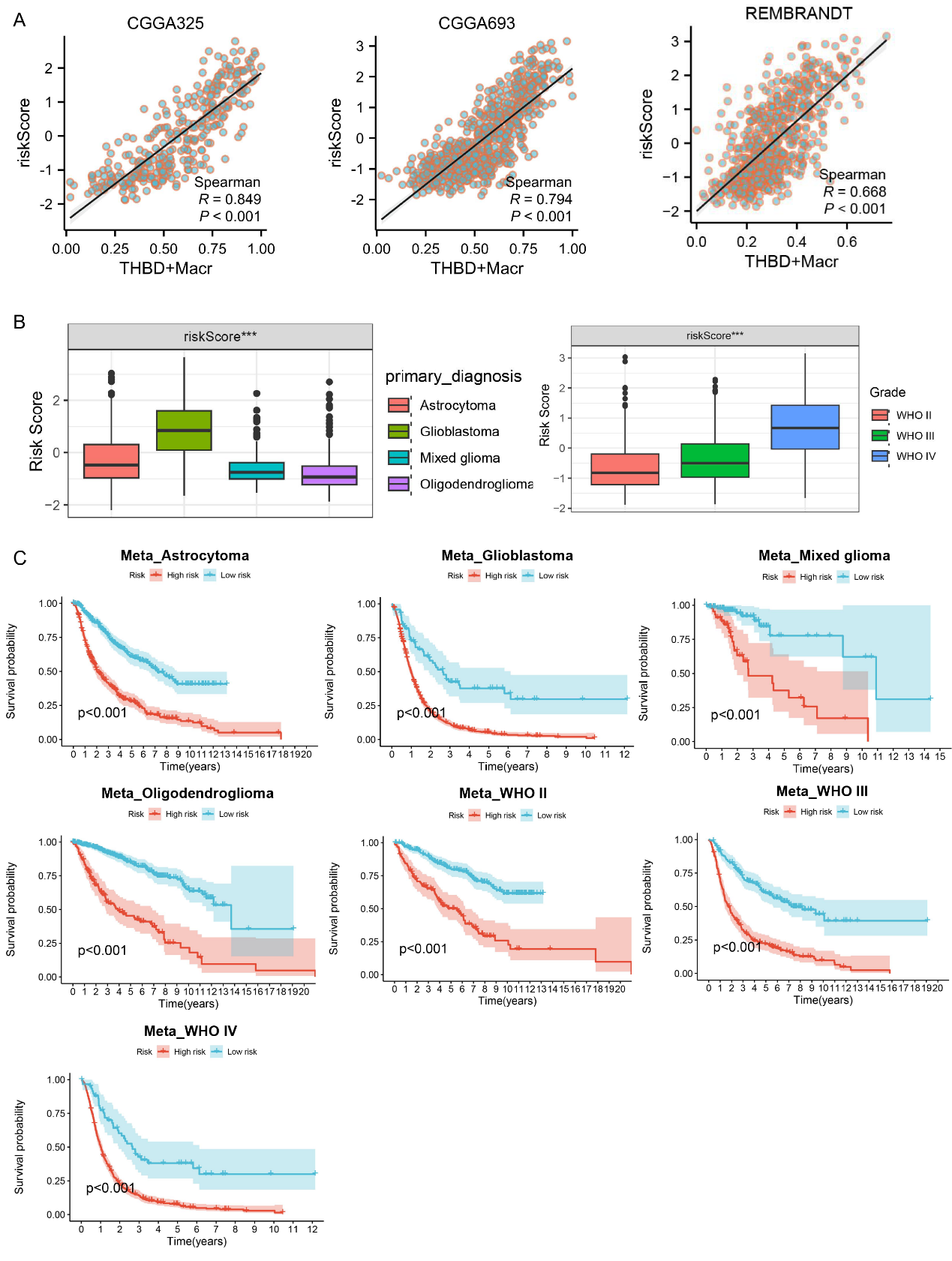


**Supplementary Figure 4. THBDMRS exhibits robust prognostic function in gliomas.**

**A.** Correlation analysis between THBD^+^ macrophage infiltration and model risk score.

**B and C.** Kaplan-Meier curves analyzing the survival of patients with high and low model risk score in different types or stages of gliomas and comparing their expression levels in the Meta_cohort dataset.


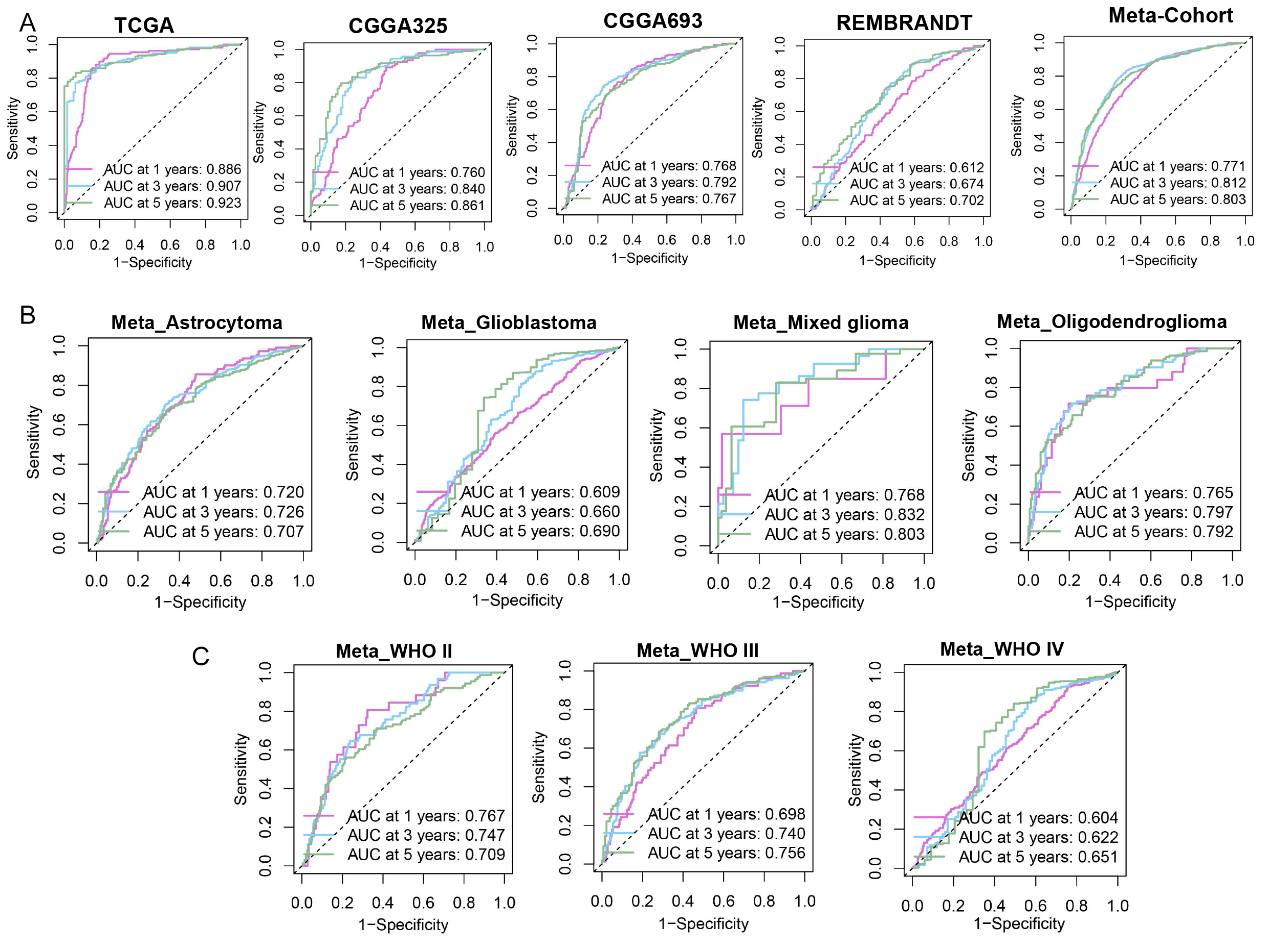


**Supplementary Figure 5. ROC curves for assessing the area under the curve (AUC) of THBDMRS in predicting 1-year, 3-year, and 5-year overall survival (OS) in glioma patients.**


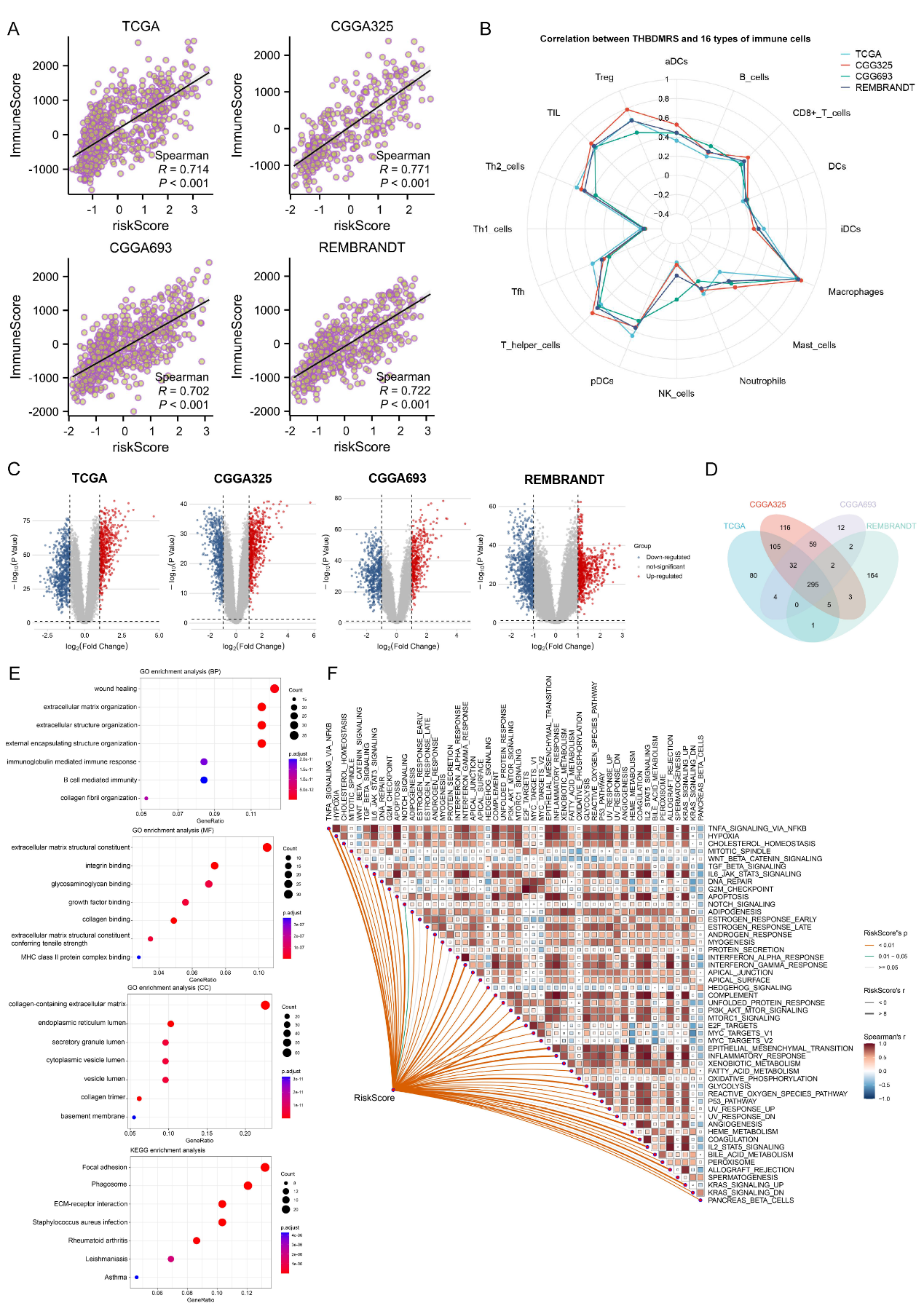


**Supplementary Figure 6. Association of THBDMRS with metastasis and extracellular matrix remodeling in glioma.**

**A.** Correlation analysis between THBDMRS risk score and immune scores. **B.** Analysis of the correlation between THBDMRS and 16 immune cell types in different datasets. **C.** Volcano plot displaying differentially upregulated genes with significant differences in various datasets. Subsequently, 295 major genes were identified after cross-analysis (**D**). These genes were further subjected to GO, KEGG (**E**), and GSVA pathway analysis (**F**).

**
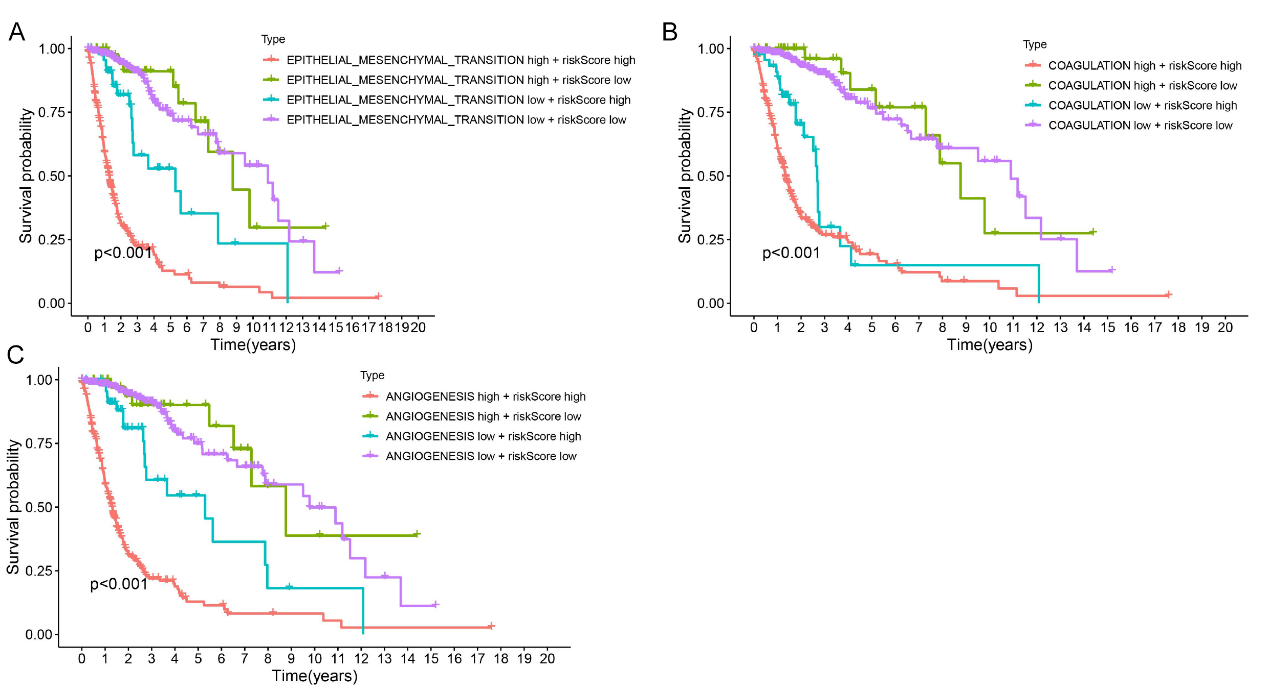
**

**Supplementary Figure 7. Kaplan-Meier curves analyzing the survival of glioma patients based on different pathways and different model risk scores.**
